# Thermochemistry and Kinetics of the Thermal Degradation of 2-Methoxyethanol as Possible Biofuel Additives

**DOI:** 10.1038/s41598-019-40890-2

**Published:** 2019-03-14

**Authors:** Mohamed A. Abdel-Rahman, Nessreen Al-Hashimi, Mohamed F. Shibl, Kazunari Yoshizawa, Ahmed M. El-Nahas

**Affiliations:** 1 Chemistry Department, Faculty of Science, Menoufia University, Shebin El-Kom, Egypt; 2 Department of Chemistry and Earth Sciences, College of Arts and Sciences, Qatar University, P.O. Box 2713 Doha, Qatar; 3 Institute for Materials Chemistry and Engineering and IRCCS, Kyushu University, Fukuoka, 819-0395 Japan

## Abstract

Oxygenated organic compounds derived from biomass (biofuel) are a promising alternative renewable energy resource. Alcohols are widely used as biofuels, but studies on bifunctional alcohols are still limited. This work investigates the unimolecular thermal degradation of 2-methoxyethanol (2ME) using DFT/BMK and ab initio (CBS-QB3 and G3) methods. Enthalpies of the formation of 2ME and its decomposition species have been calculated. Conventional transition state theory has been used to estimate the rate constant of the pyrolysis of 2ME over a temperature range of 298–2000 K. Production of methoxyethene via 1,3-H atom transfer represents the most kinetically favored path in the course of 2ME pyrolysis at room temperature and requires less energy than the weakest C_α_ − C_β_ simple bond fission. Thermodynamically, the most preferred channel is methane and glycoladhyde formation. A ninefold frequency factor gives a superiority of the C_α_ − C_β_ bond breaking over the C_γ_ − O_β_ bond fission despite comparable activation energies of these two processes.

## Introduction

Limited energy reserves and global environmental impact of fossil fuel burning became a crucial issue pushing to searching for alternative renewable sources of energy^1–6^. Biofuels represent a promising alternative renewable source of energy. Biofuels appear in the energy map of many industrial countries^7,8^. Therefore, a revolution occurred in the forums of the production of biofuels from different biomasses.

Among biofuels, the most popular bioethanol suffers from some drawbacks such as low internal energy, water absorption, very high ignition temperature, lower combustion efficiency, and high vapor pressure causing massive emissions to the atmosphere^8–10^ giving rise to adverse effects on the human health^11^. In order to avoid most of the above issues, bigger oxygenated materials are preferred. For instance, 2-methoxyethanol (2ME) with bifunctional groups namely etheric (O) and hydroxyl group (OH) is proposed as a model for sizeable molecular biodiesel additive hydroxyethers^12^ since it can mimic the behavior of the latter in the combustion process. Furthermore, 2ME is an excellent indirect biofuel candidate due to its original synthesis from small bioalcohols like methanol and ethanol. Besides, it can be obtained by modifying ethylene glycol (EG) itself. Ethylene glycol had recently become available from different biomass categories using various procedures with high yield^13–19^ as a biofuel, but there still some concerns related to its low carbon content, low melting point (−13 °C), high viscosity, high toxicity, and high hydrophilic nature^20^. Those issues can be avoided by using alone in the current engine infrastructure. 2ME could function as a biofuel that might be better than ethanol, ethylene glycol regarding lower vapor pressure, higher boiling point, and high energy content. It also shows high miscibility with oils and gasoline besides the expected enhanced ignition behavior due to its high oxygen content (42.1% per mol). These represent some essential useful properties for 2ME as a good biofuel candidate.

2ME has a wide range for applications in industrial and pharmaceutical proposes. For instance, it is used in inks, resins, dyes, paints, metal coatings, phenolic varnishes, detergents, cosmetics, cleaners’ products, protective coatings like lacquers, and in airplane fuels as anti-freezing agent^21,22^. To the best of our knowledge, there is neither experimental nor theoretical work related to 2ME as a biofuel candidate. Therefore, we are going to shed new light on this subject in an attempt to explore the thermochemistry and kinetics of 2ME pyrolysis as biofuel additives. The current study could guide interpretation of the future experimental data obtained from 2ME combustion. The Focal point analysis (FPA)^23–26^ is a highly effective method of the modern high *ab initio* theory which closely related to Wn (n = 1–3)^27^ and HEATn (High accuracy Extrapolated Ab initioThermochemistry) methods^28^. FPA showed good results when applied to small to moderate size compounds^23–26,29^.

This paper is organized as follows: Section 2 covers computational methods details. Section 3 presents the results and discussion which is divided into subsections for 2ME conformers, bond dissociation energies, enthalpy of formation, energies and IRC analysis, and rate constant calculation. Finally, section 4 concludes.

### Computational details

Geometry optimization for 2ME, its decomposition products, and transition states have been performed using density functional theory (DFT) employing the Bose-Martin functional developed for kinetics (BMK)^30^ (42% electron correlation) in conjunction with the 6–31+G(d,p) basis set. The multi-level complete basis set CBS-QB3^31–33^ and G3^33^ ab initio methods have also been used for more accurate energies calculations at a moderate computational cost. The expensive W1 method which is the first of Wn^34,35^ series is used. Wn (n = 1–3) methods are more accurate and expensive than CBS-QB3 and G3 methods and recommend for small systems investigations^34,35^.

Focal-point analysis approximation has been applied to determine the relative energies of both most stable and least stable 2ME conformers. The highly accurate energy difference is obtained by single point calculations at CCSD(T)/aug-cc-pVTZ, MP3/aug-cc-pVQZ, and MP2/aug-cc-pV5Z levels based on optimized structures of the selected conformers at B3LYP/aug-cc-pVTZ level. A three-parameter exponential formula was used for Hartree-Fock (HF) energy extrapolation to the complete basis set (CBS):

1 $${{\rm{E}}}_{{\rm{X}}}^{{\rm{HF}}}={{\rm{E}}}_{{\rm{CBS}}}^{{\rm{HF}}}+{{\rm{ae}}}^{-{\rm{bx}}}$$

where X = {T, Q, 5} for aug-cc-pVTZ, aug-cc-pVQZ, and aug-cc-pV5Z, respectively^36^. The extrapolation of the MP2 was obtained by the two-parameter polynomial equation:

2 $${{\rm{E}}}_{{\rm{X}}}^{{\rm{MP}}2}={{\rm{E}}}_{{\rm{CBS}}}^{{\rm{MP}}2}+{{\rm{aX}}}^{-3}$$

where X = {Q, 5} for aug-cc-pVQZ and aug-cc-pV5Z, respectively.

The transition states for different reactions of 2ME pyrolysis have been located with the aid of the eigenvector-following (EF) optimization technique as implemented in the Gaussian programs. Vibrational analyses have been conducted at BMK/6–31+G(d, p) to characterize the nature of the obtained stationary points whether they are minima or transition states with real frequencies or one imaginary frequency, respectively, and for the zero-point vibrational as well as the thermal corrections of energies at 298 K. Vibrational modes have been analyzed using the Chemcraft program^37^. For further confirmation of correct transition states that connect desired reactants and products, minimum energy paths (MEP) have been computed through intrinsic reaction coordinates (IRC)^38,39^. All electronic structures calculations have been conducted using the Gaussian 09 W suite of programs^40^.

The atomization energy approach has been exploited to estimate the gas phase enthalpies of formations for 2ME and its released species at the standard state of temperature and pressure, as it is deduced from the well-known^41^ enthalpies of formation of the separated atoms. For any molecule M containing X numbers of isolated atoms, the gas phase enthalpy of formation is obtained from

$$\begin{array}{rcl}{\rm{\Delta }}{H}_{{\rm{f}}}^{^\circ }{\rm{gas}}({\rm{M}}) & = & {E}_{e}({\rm{M}})+{\rm{ZPVE}}({\rm{M}})\\  &  & +\,[{H}_{298}({\rm{M}})-{H}_{0}({\rm{M}})]-\sum _{{\rm{i}}}^{{\rm{atoms}}}\{{E}_{{\rm{e}}}({{\rm{X}}}_{{\rm{i}}})\\  &  & +\,[{H}_{298}({{\rm{X}}}_{{\rm{i}}})-{H}_{0}({{\rm{X}}}_{{\rm{i}}})]\}+\sum _{{\rm{i}}}^{{\rm{atoms}}}{\rm{\Delta }}{H}_{{\rm{f}}}^{^\circ }{\rm{gas}}({{\rm{X}}}_{{\rm{i}}}),\end{array}$$

where *E*_e_ (M) and *E*_e_ (X_i_) are the theoretically calculated electronic energy of molecule M, and the i^th^ atom X at the same level of theory, respectively. ZPVE is the zero-point vibrational energy of the molecule. [*H*_298_(M) − *H*_0_(M)] and [*H*_298_(X_i_) − *H*_0_(X_i_)] are thermal corrections to the enthalpy for the molecule M and the separated atoms X, respectively. The individual atomic enthalpies Δ*H*^°^_f_(X_i_) are extracted from the NIST WebBook^41^. Kinetic parameters for different channels of 2ME pyrolysis have been estimated over a wide range of temperatures using the Kisthelp package program^42^, where the classical transition state theory (TST)^43^ is coupled with Eckart tunneling correction^44^ to compute rate constants (*k*) for H-atom transfer reactions of 2ME pyrolysis over the applied range of temperatures (298–2000 K). The rate constant reads:

$${k}^{{\rm{T}}{\rm{S}}{\rm{T}}}(T)=\chi (T)\sigma \frac{{k}_{{\rm{B}}}T}{h}{(\frac{{\rm{R}}T}{{{\rm{p}}}^{\circ }})}^{{\rm{\Delta }}n}{e}^{-{{\rm{\Delta }}}^{\ddagger }{G}^{\circ }(T)/{{\rm{k}}}_{{\rm{B}}}T},$$

where *h*, *k*_B_, and R symbols are Planck, Boltzmann, and universal ideal gas constants, respectively, and ${\rm{\chi }}(T)$ is the Eckart tunneling correction. *T* is the system’s temperature in Kelvin, *σ* is reaction path degeneracy, p° is the standard pressure (1 atm), and ${{\rm{\Delta }}}^{\dagger }{G}^{^\circ }(T)$ is the standard Gibbs free energy of activation for reaction. Δn takes two value either zero in the case of unimolecular decomposition or 1 in the case of the bimolecular oxidation.

The more accurate correction term Eckart tunneling correction χ(*T*) which obtained through the numerical integrating probability of transmission ρ(*E*) over Boltzmann distribution of energies. The asymmetric Eckart tunneling correction gives reliable results at low temperatures and previously demonstrated in many previous publications^45–47^.

The transmission probability coefficient χ(T) can deduce from the following equation:

$${\rm{\chi }}({\rm{T}})=\frac{{{\rm{e}}}^{\frac{{{\rm{\Delta }}H}_{{\rm{f}}}^{0{\rm{K}}}}{{{\rm{k}}}_{{\rm{B}}}{\rm{T}}}}}{{{\rm{k}}}_{{\rm{B}}}{\rm{T}}}\,{\int }_{0}^{\infty }\,{\rm{\rho }}({\rm{E}}){{\rm{e}}}^{-\frac{{\rm{E}}}{{{\rm{K}}}_{{\rm{B}}}{\rm{T}}}}{\rm{dE}},$$

where ρ(*E*) is the probability of transmission through the one-dimensional barrier at energy E. ${{\rm{\Delta }}H}_{{\rm{f}}}^{0{\rm{K}}}$ is the zero point correlated energy barriers in the forward direction.

Equilibrium relation (*K*_eq_ = *k*_forward_/*k*_reverse_) has been used to calculate the rate constant of simple fission reactions. At first, the equilibrium constant (*K*_eq_) was calculated automatically by the assistance of the Kisthelp program^42^ then the previous experimental well-known association rate constants^48–50^ have been used as values for *k*_reverse_ to get the forward rate constants (*k*_forward_) for the selected simple bond fission reaction.

All complex fission reactions barrier heights have been investigated using the more accurate *ab initio* CBS-QB3, G3, and BMK/6–31+G (d, p). The last level of theory has been proven to have a significant efficiency for the structure optimization in previous works^51,52^.

## Results and Discussion

### Methoxyethanol conformers

**Figure 1:**
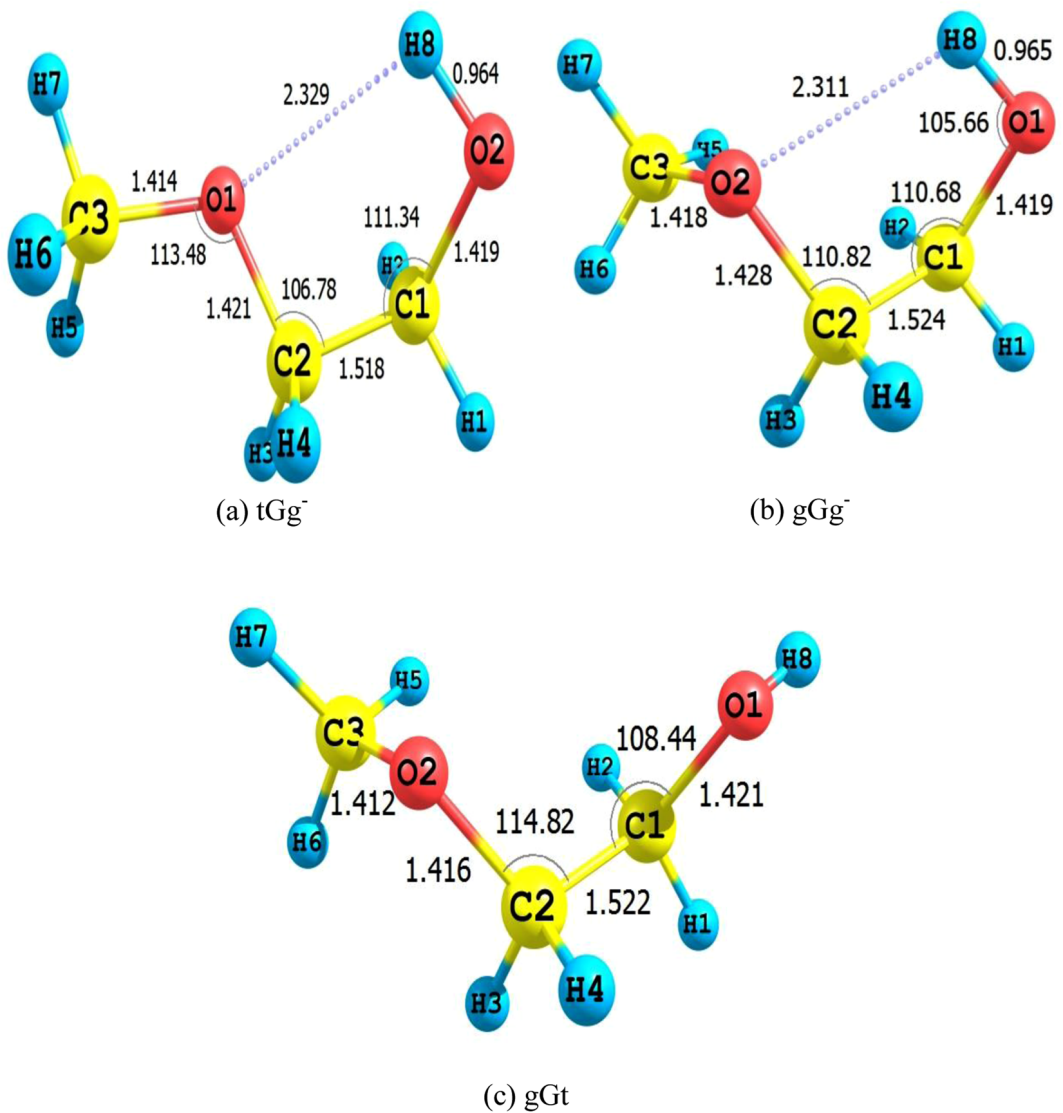
Optimized structures of 2ME conformers at B3LYP/6–311G(d, p) (optimization level of CBS-QB3).

**Figure 2:**
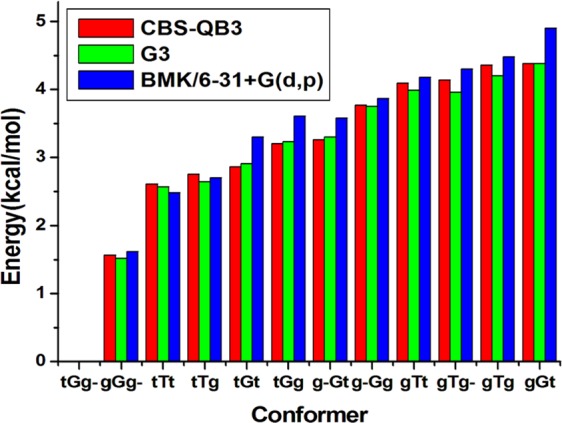
Relative stabilities of 2ME conformers (energy calculated relative to tGg^−^) at CBS-QB3, G3, and BMK/6–31+G (d, p).

2ME has 12 conformers. Three of them are illustrated in Fig. 1, and the rest of the optimized structures and energies are presented in the Supporting Information (SI). Several studies on 2ME conformers highlighted the effect of the intramolecular hydrogen bond (IHB) between the alcoholic hydrogen and etheric oxygen on molecular properties^53–56^. Our findings at CBS-QB3, G3, and BMK/6–31+G(d, p) are in mutual harmony, see Fig. 2. The most stable 2ME conformers adopt tGg^−^ and gGg^−^ structures with IHB^54–56^. However, tGg^−^ is 1.6 kcal/mol more stable than gGg^−^. On the other hand, the least stable conformer (gGt), among the studied conformers, is 4.38 kcal/mol higher than tGg^−^ at the CBS-QB3 level of theory.

### Energy of 2ME conformations: Extrapolation to CCSD(T)/CBS level using FPA

**Figure 3:**
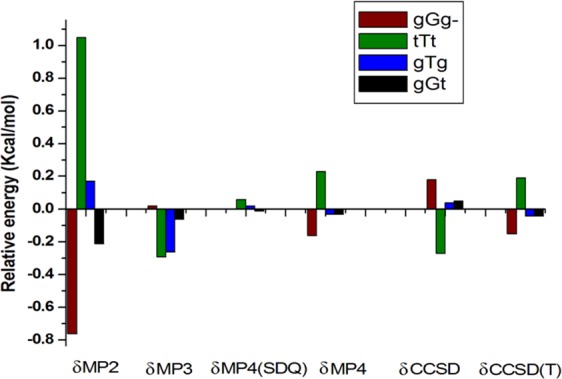
Focal point analysis results for aug-cc-pVTZ basis set.

Tables 1 and 2 collect the results of FPA for the most and least stable 2ME conformers, while Table 3 shows a comparison of FPA results at MP2/CBS and CCSD(T)/CBS with our obtained values using *ab initio* methods (CBS-QB3 and G3) and the DFT/BMK/6–31+G(d,p). The CCSD(T)/CBS energies are 1.43 ± 0.15, 2.47 ± 0.19, 4.11 ± 0.04, and 4.25 ± 0.04 for the conformers gGg-, tTt, gTg, and gGt, respectively. The uncertainty term is obtained using δ [CCSD(T)] ± ∆E_CCSD(T)_ − ∆E_CCSD_. Comparing the obtained results of the FPA with that of our 2ME conformation analysis using *ab initio* composite methods and BMK/6–31+G(d,p) level shows harmony as appeared in Table 3, while the convergence of the quantum chemical electron correlations methods at the aug-cc-pVTZ basis set is sketched in Fig. 3.

**Table 1:** The valence focal-point analysis (FPA) of energy differences (kcal/mol) of the most stable 2ME conformers (a) gGg- and (b) tTt.

	∆E_HF_	δ[MP2]	δ [MP3]	δ [MP4(SDQ)]	δ [MP4]	δ [CCSD]	δ [CCSD(T)]	∆E_CCSDT_
**(a) gGg** ^−^								
aCCD	2.13	−0.74	0.01	0.01	−0.13	0.14	−0.14	1.28
aCCT	2.26	−0.76	0.02	0.00	−0.16	0.18	−0.15	1.38
aCCQ	2.27	−0.74	0.03	[0.00]	[−0.16]	[0.18]	[−0.15]	[1.43]
aCC5	2.27	−0.74	[0.03]	[0.00]	[−0.16]	[0.18]	[−0.15]	[1.43]
CBS	2.27	−0.74	[0.03]	[0.00]	[−0.16]	[0.18]	[−0.15]	[**1**.**43**]
**(b) tTt**								
aCCD	1.58	1.00	−0.28	0.06	0.20	−0.24	0.17	2.49
aCCT	1.50	1.05	−0.29	0.06	0.23	−0.27	0.19	2.48
aCCQ	1.49	1.04	−0.29	[0.06]	[0.23]	[−0.27]	[0.19]	[2.46]
aCC5	1.49	1.06	[−0.29]	[0.06]	[0.23]	[−0.27]	[0.19]	[2.46]
CBS	1.48	1.07	[−0.29]	[0.06]	[0.23]	[−0.27]	[0.19]	[**2**.**47**]

Conformer geometries have been optimized at B3LYP/aug-cc-pVTZ level. aCCD = aug-cc pVDZ; aCCT = aug-cc-pVTZ; aCCQ = aug-cc-pVQZ; aCC5 = aug-cc-pV5Z; CBS = complete basis set. The symbol δ denotes the increment in the relative energy concerning the previous level of theory, as given by the competing higher-order correlation series: HF → MP2 → MP3 → MP4(SDQ) → MP4 → CCSD → CCSD(T). For example, δ [MP4] = ∆E_MP4_ − ∆E_MP4(SDQ)_. Values listed in brackets are taken for extrapolation. Equations (1) and (2) have been used for extrapolation of HF and MP2 energies to complete the basis set, respectively. Final values (in bold) include core correction.

**Table 2:** The valence focal-point analysis (FPA) of energy differences (kcal/mol) of the least stable 2ME conformers (a) gTg and (b) gGt.

	∆E_HF_	δ[MP2]	δ [MP3]	δ [MP4(SDQ)]	δ [MP4]	δ [CCSD]	δ [CCSD(T)]	∆E_CCSDT_
**(a) gTg**								
aCCD	4.11	0.10	−0.26	0.04	−0.03	0.02	−0.04	3.94
aCCT	4.13	0.17	−0.26	0.02	−0.03	0.04	−0.04	4.04
aCCQ	4.12	0.20	−0.24	[0.02]	[−0.03]	[0.04]	[−0.04]	[4.08]
aCC5	4.12	0.22	[−0.24]	[0.02]	[−0.03]	[0.04]	[−0.04]	[4.09]
CBS	4.12	0.23	[−0.24]	[0.02]	[−0.03]	[0.04]	[−0.04]	[**4**.**11**]
**(b) gGt**								
aCCD	4.53	−0.35	−0.06	−0.01	−0.04	0.06	−0.04	4.09
aCCT	4.49	−0.21	−0.06	−0.01	−0.03	0.05	−0.04	4.19
aCCQ	4.49	−0.19	−0.05	[−0.01]	[−0.03]	[0.05]	[−0.04]	[4.23]
aCC5	4.49	−0.17	[−0.05]	[−0.01]	[−0.03]	[0.05]	[−0.04]	[4.24]
CBS	4.49	−0.16	[−0.05]	[−0.01]	[−0.03]	[0.05]	[−0.04]	[**4**.**25**]

See comments in Table 1.

**Table 3:** Comparison of FPA results with other computational methods.

Conformer	FPA		BMK/6–31+G(d, p)	CBS-QB3	G3
	CCSD(T)/CBS	MP2/CBS			
tGg^−^	0	0	0	0	0
gGg^−^	1.43	1.53	1.62	1.57	1.52
tTt	2.47	2.55	2.48	2.61	2.57
gTg	4.11	4.35	4.48	4.36	4.20
gGt	4.25	4.33	4.90	4.38	4.38

### Bond dissociation energy

**Figure 4:**
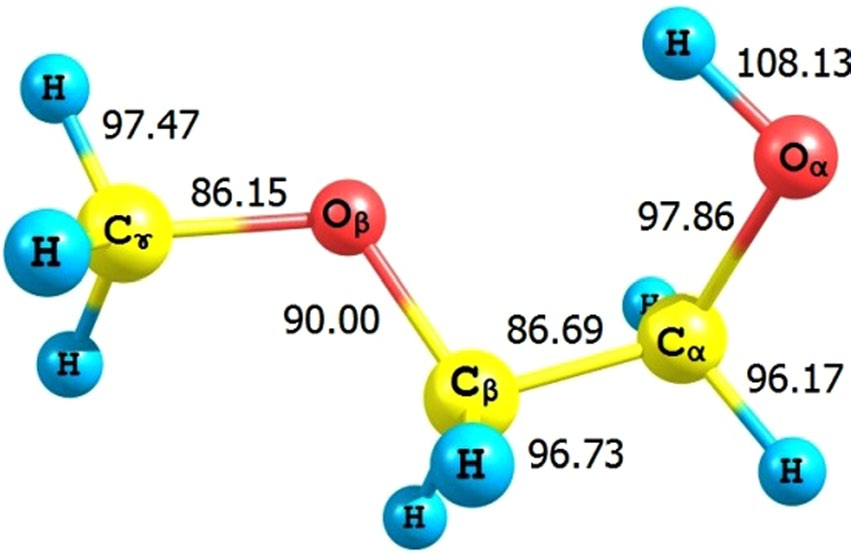
Bond dissociation energies (BDE) of 2ME (kcal/mol) at CBS-QB3 at room temperature.

In order to assess the strengths of different bonds in 2ME, their bond dissociation energies have been calculated. Figure 4 displays the bond dissociation energies of 2ME using the CBS-QB3 composite method. The results indicate that the C_ɤ_−O_β_ and C_α_−C_β_ are the weakest bonds with bond dissociation energies of 86.2 and 86.7 kcal/mol, respectively. The alcoholic O_α_-H bond is the strongest one which is close to our previous results (104.5–106.3 kcal/mol) obtained for C1-C4 alcohols^52,57^. The C_α_-H and C_β_-H hydrogen atoms are the most acidic and are expected to be abstracted easier in the presence of oxidizing agents as compared to the other hydrogen atoms which agreed with similar bifunctional compound^58^.

### Enthalpies of Formation

Enthalpies of formation for 2ME and its released compounds through combustion have been calculated using atomization approach (at CBS-QB3) and isodesmic equations procedures (at BMK/6–31+G (d, p)). The obtained results are collected in Table 4 using experimental enthalpies of formation values of Table 5. The results have been compared with one another and with available experimental data. The comparison shows impressive agreement with a maximum deviation of ±2 kcal/mol which gives confidence in the future experimental determination of unknown species.

**Table 4:** Enthalpies of formation for 2ME, and its relevant compounds calculated by using atomization energies approach (AE) at CBS-QB3 and isodesmic reactions at BMK/6–31+G (d, p), results are in Kcal/mol.

Species	AE	Isodesmic	Exp.
CH_3_OCH_2_CH_2_OH	−91.29	−90.21	−90.04 ± 1.94^a^, −94.58^b^
CH_3_OCH_2_OCH_3_	−83.67	−81.96	−83.18 ± 0.19^c^
HOCH_2_CH_2_OH	−94.76	−89.85	−92.57^d^, −93^e^, −93.24^f^, −92.69^g^, −94.22 ± 0.67^h^
CH_3_OCH_2_CHO	−68.68	−66.56	
OHCCH_2_OH	−72.17	−71.02	
CH_3_OCH=CH_2_	−26.02	−26.88	
Cyc(C_3_H_6_O)	−19.57		−19.24^i^, −19.24 ± 0.15^j^
CH_2_=CHOH	−28.67	−30.11	−30.57^k^, −29.86 ± 2^l^, −26.52 ± 2^m^
CH_3_OCH_3_	−45.39	−44.94	−44 ± 0.12^n^
CH_3_OH	−48.88	−47.89	−48.99 ± 2.39^o^
CH_3_OCH_2_CH_2_O^∙^	−35.26	−34.21	
CH_3_OCH_2_CH^∙^OH	−47.22	−47.23	
CH_3_OCH^∙^CH_2_OH	−46.67	−46.50	
^∙^CH_2_OCH_2_CH_2_OH	−45.91	−46.64	
HOCH_2_CH_2_O^∙^	−40.61	−42.14	
CH_3_OCH_2_CH_2_^∙^	−2.23	−2.40	
CH_3_OCH_2_^∙^	−0.47	−1.07	−0.10^p^
CH_3_O^∙^	4.27	5.77	5.02 ± 0.50^q^, 4.06 ± 0.96^r^
^∙^∙CH_2_OH	−4.12	−5.14	−3.97 ± 0.22^s^, −2.15 ± 0.95^r^

^a^References^65^, ^b^ref. ^66^, ^c^ref. ^67^, ^d^ref. ^68^, ^e^ref. ^69^, ^f^ref. ^70^, ^g^ref. ^71^, ^h^ref. ^72^, ^i^ref. ^73^, ^j^ref. ^74^, ^k^ref. ^75^, ^l^ref. ^76^, ^m^ref. ^77^, ^n^ref. ^78^, ^o^ref. ^79^, ^p^ref. ^80^, ^q^ref. ^81^, ^r^ref. ^82^, ^s^ref. ^83^.

**Table 5:** Experimental Enthalpies of formation for reference species used in isodesmic reactions.

Species	Exp.	Refs	Species	Exp.	Refs
CH_3_CH_2_CH_2_CH_2_OH	−65.70	^84^	CH_3_^∙^CHOH	−14.50 ± 3	^85^
CH_3_CH_2_CH_2_OH	−60.97	^86^	CH_3_CH_2_CH_2_O^∙^	−9.90	^87^
CH_3_CH_2_OH	−56.12 ± 0.12	^88^	^∙^CH_2_CH_2_OH	−7.00	
CH_3_OH	−48.06 ± 0.05	^79^	^∙^CH_2_OH	−4.09 ± 0.81	^89^
CH_3_OCH_3_	−44 ± 0.12	^78^	CH_3_O^∙^CH_2_	−0.10	^80^
CH_3_CH_2_OCH_3_	−51.72 ± 0.16	^80^	CH_3_CH_2_CH = CH_2_	−0.15 ± 0.19	^90^
CH_3_OCH_2_CH_2_OCH_3_	−81.93 ± 0.17	^91^	CH_3_CH = CH_2_	4.88	^92^
CH_3_CH_3_	−20.03 ± 0.07	^93^	CH_3_^∙^CH_2_	28.39 ± 0.31	^94^
CH_3_CH_2_CH_3_	−24.90 ± 0.12	^93^	CH_3_CH_2_^∙^CH_2_	23.9 ± 0.48	^82^
CH_3_CH(CH_3_)_2_	−32.07 ± 0.15	^93^	^∙^CH_2_CH(CH_3_)_2_	16.73	^82^
HCOOH	−90.49	^95^	HCHO	−26.05 ± 0.43	^96^
HOCH_2_CH_2_OH	−92.57 ± 0.48	^97^	CH_4_	−17.89	^98^
HOCH_2_CH_2_CH_2_OH	−97.54 ± 1.22	^71^	CH_3_O^∙^	5.02 ± 0.50	^81^
(CH_3_)_2_CHOH	−65.20	^84^	(CH_3_)_2_CHO^∙^	−11.10 ± 1.20	^99^
CH_3_CHO	−39.70 ± 0.12	^100^	^∙^CH_2_CHO	3.51 ± 0.38	^101^
C_2_H_5_CHO	−45.08 ± 0.19	^102^	CH_3_OCHO	−86.47	^103^
CH_3_CH_2_OOH	−41.92 ± 3.08	^104^	CH_3_CH_2_OO^∙^	−6.55 ± 2.37	^104^
CH_3_OOH	−31.31	^105^	CH_3_CH_2_O^∙^	−3.25 ± 0.96	^81^
CH_2_ = CH_2_	12.55	^98^	CH_3_CH_2_COOCH_2_CH_3_	−111.81	^106^
CH_2_ = CH-CH_2_OH	−29.53 ± 0.36	^97^	CH_3_^∙^CHCOOCH_2_CH_3_	−68.83	^106^
CH_2_ = CH-OH	−30.59	^75^			

The current study concentrates on 2ME pyrolysis. The decomposition mechanism can be expanded into nine complex fissions (barrier reactions) and eight simple bond scission reactions (barrierless reactions).

Complex fission reactions

R1 $${{\rm{CH}}}_{{\rm{3}}}{{\rm{OCH}}}_{{\rm{2}}}{{\rm{CH}}}_{{\rm{2}}}{\rm{OH}}\to {\rm{TS1}}\to {{\rm{CH}}}_{3}{\rm{OCH}}={{\rm{CH}}}_{2}+{{\rm{H}}}_{{\rm{2}}}{\rm{O}}$$

R2 $${{\rm{CH}}}_{{\rm{3}}}{{\rm{OCH}}}_{{\rm{2}}}{{\rm{CH}}}_{{\rm{2}}}{\rm{OH}}\to {\rm{TS}}2\to {{\rm{CH}}}_{{\rm{3}}}{\rm{OH}}+{{\rm{CH}}}_{2}={\rm{CHOH}}$$

R3 $${{\rm{CH}}}_{{\rm{3}}}{{\rm{OCH}}}_{{\rm{2}}}{{\rm{CH}}}_{{\rm{2}}}{\rm{OH}}\to {\rm{TS}}3\to {{\rm{CH}}}_{3}{{\rm{OCH}}}_{2}^{\cdot \cdot }{\rm{CH}}+{{\rm{H}}}_{{\rm{2}}}{\rm{O}}$$

R4 $${{\rm{CH}}}_{{\rm{3}}}{{\rm{OCH}}}_{{\rm{2}}}{{\rm{CH}}}_{{\rm{2}}}{\rm{OH}}\to {\rm{TS}}4\to {\rm{HCHO}}+{{\rm{C}}}_{{\rm{2}}}{{\rm{H}}}_{{\rm{5}}}{\rm{OH}}$$

R5 $${{\rm{CH}}}_{{\rm{3}}}{{\rm{OCH}}}_{{\rm{2}}}{{\rm{CH}}}_{{\rm{2}}}{\rm{OH}}\to {\rm{TS}}5\to {{\rm{HOCH}}}_{2}{{\rm{CH}}}_{2}{\rm{OH}}+{}^{\cdot \cdot }{\rm{C}}{{\rm{H}}}_{2}$$

R6 $${{\rm{CH}}}_{{\rm{3}}}{{\rm{OCH}}}_{{\rm{2}}}{{\rm{CH}}}_{{\rm{2}}}{\rm{OH}}\to {\rm{TS}}6\to {{\rm{CH}}}_{{\rm{3}}}{{\rm{OCH}}}_{{\rm{2}}}{\rm{CHO}}+{{\rm{H}}}_{2}$$

R7 $${{\rm{CH}}}_{{\rm{3}}}{{\rm{OCH}}}_{{\rm{2}}}{{\rm{CH}}}_{{\rm{2}}}{\rm{OH}}\to {\rm{TS}}7\to {{\rm{CH}}}_{{\rm{3}}}{{\rm{OCH}}}_{3}+{\rm{HCHO}}$$

R8 $${{\rm{CH}}}_{{\rm{3}}}{{\rm{OCH}}}_{{\rm{2}}}{{\rm{CH}}}_{{\rm{2}}}{\rm{OH}}\to {\rm{TS}}8\to {\rm{Cyc}}({{\rm{C}}}_{{\rm{3}}}{{\rm{H}}}_{{\rm{6}}}{\rm{O}})+{{\rm{H}}}_{{\rm{2}}}{\rm{O}}$$

R9 $${{\rm{CH}}}_{{\rm{3}}}{{\rm{OCH}}}_{{\rm{2}}}{{\rm{CH}}}_{{\rm{2}}}{\rm{OH}}\to {\rm{TS}}9\to {{\rm{CH}}}_{4}+{{\rm{OHCCH}}}_{{\rm{2}}}{\rm{OH}}$$

Simple bond fission reactions

R10 $${{\rm{CH}}}_{{\rm{3}}}{{\rm{OCH}}}_{{\rm{2}}}{{\rm{CH}}}_{{\rm{2}}}{\rm{OH}}\to {}^{\cdot }{\rm{C}}{{\rm{H}}}_{3}+{}^{\cdot }{\rm{O}}{{\rm{CH}}}_{{\rm{2}}}{{\rm{CH}}}_{{\rm{2}}}{\rm{OH}}$$

R11 $${{\rm{CH}}}_{{\rm{3}}}{{\rm{OCH}}}_{{\rm{2}}}{{\rm{CH}}}_{{\rm{2}}}{\rm{OH}}\to {{\rm{CH}}}_{{\rm{3}}}{{\rm{O}}}^{\cdot }+{}^{\cdot }{\rm{C}}{{\rm{H}}}_{{\rm{2}}}{{\rm{CH}}}_{{\rm{2}}}{\rm{OH}}$$

R12 $${{\rm{CH}}}_{{\rm{3}}}{{\rm{OCH}}}_{{\rm{2}}}{{\rm{CH}}}_{{\rm{2}}}{\rm{OH}}\to {{\rm{CH}}}_{{\rm{3}}}{{\rm{O}}}^{\cdot }{{\rm{CH}}}_{2}+{}^{\cdot }{\rm{C}}{{\rm{H}}}_{{\rm{2}}}{\rm{OH}}$$

R13 $${{\rm{CH}}}_{{\rm{3}}}{{\rm{OCH}}}_{{\rm{2}}}{{\rm{CH}}}_{{\rm{2}}}{\rm{OH}}\to {{\rm{CH}}}_{{\rm{3}}}{{\rm{OCH}}}_{2}^{\cdot }{{\rm{CH}}}_{2}+{}^{\cdot }{\rm{O}}{\rm{H}}$$

R14 $${{\rm{CH}}}_{{\rm{3}}}{{\rm{OCH}}}_{{\rm{2}}}{{\rm{CH}}}_{{\rm{2}}}{\rm{OH}}\to {{\rm{CH}}}_{{\rm{3}}}{{\rm{OCH}}}_{{\rm{2}}}{{\rm{CH}}}_{{\rm{2}}}{{\rm{O}}}^{\cdot }+{{\rm{H}}}^{\cdot }$$

R15 $${{\rm{CH}}}_{{\rm{3}}}{{\rm{OCH}}}_{{\rm{2}}}{{\rm{CH}}}_{{\rm{2}}}{\rm{OH}}\to {}^{\cdot }{\rm{C}}{{\rm{H}}}_{{\rm{2}}}{{\rm{OCH}}}_{{\rm{2}}}{{\rm{CH}}}_{{\rm{2}}}{\rm{OH}}+{{\rm{H}}}^{\cdot }$$

R16 $${{\rm{CH}}}_{{\rm{3}}}{{\rm{OCH}}}_{{\rm{2}}}{{\rm{CH}}}_{{\rm{2}}}{\rm{OH}}\to {{\rm{CH}}}_{{\rm{3}}}{{\rm{OCH}}}^{\cdot }{{\rm{CH}}}_{{\rm{2}}}{\rm{OH}}+{{\rm{H}}}^{\cdot }$$

R17 $${{\rm{CH}}}_{{\rm{3}}}{{\rm{OCH}}}_{{\rm{2}}}{{\rm{CH}}}_{{\rm{2}}}{\rm{OH}}\to {{\rm{CH}}}_{{\rm{3}}}{{\rm{OCH}}}_{2}^{\cdot }{\rm{CHOH}}+{{\rm{H}}}^{\cdot }$$

Complex fission reactions are those reactions proceeding by H-atom transfers via cyclic transition state, while simple bonds fission are those occurring by homolytic cleavage of the chemical bonds. We will concentrate here on that formed due to complex ones. Among nine unimolecular complex reactions, the formation of methoxyethene, methoxy methylcarbene, and oxetane occurs by dehydration (R1, R3, and R8), while 2-methoxy acetaldehyde is formed via hydrogen molecule elimination (R6) reactions. Reaction R5 proceeds via three-membered ring transition state producing ethylene glycol and triplet methylene. The other complex fission reactions R2, R4, R7, and R9 are accomplished by 1,3-H atom transfer reactions via four-membered ring transition state to produce methanol and vinyl alcohol, formaldehyde and ethanol, formaldehyde and dimethyl ether, and methane and glycolaldehyde, respectively.

**Figure 5:**
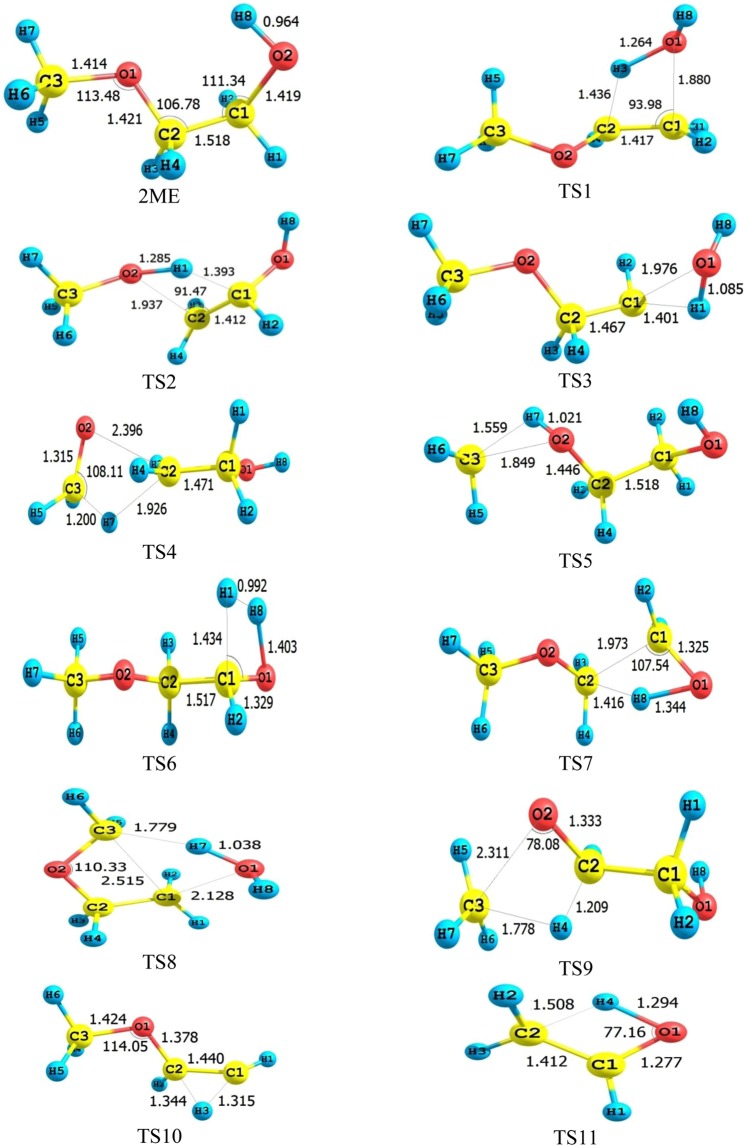
Optimized geometrical structure of 2ME and transition states for its thermal degradation at CBS-QB3.

The optimized structure of 2ME and proposed transition states leads to the formation of methoxyethene (TS1), vinyl alcohol (TS2), methoxymethyl carbene (TS3), ethanol (TS4), ethylene glycol (TS5), 2-methoxy acetaldehyde (TS6), dimethyl ether (TS7), oxetane (TS8), and glycolaldehyde (TS9) given in Fig. 5.

**Figure 6:**
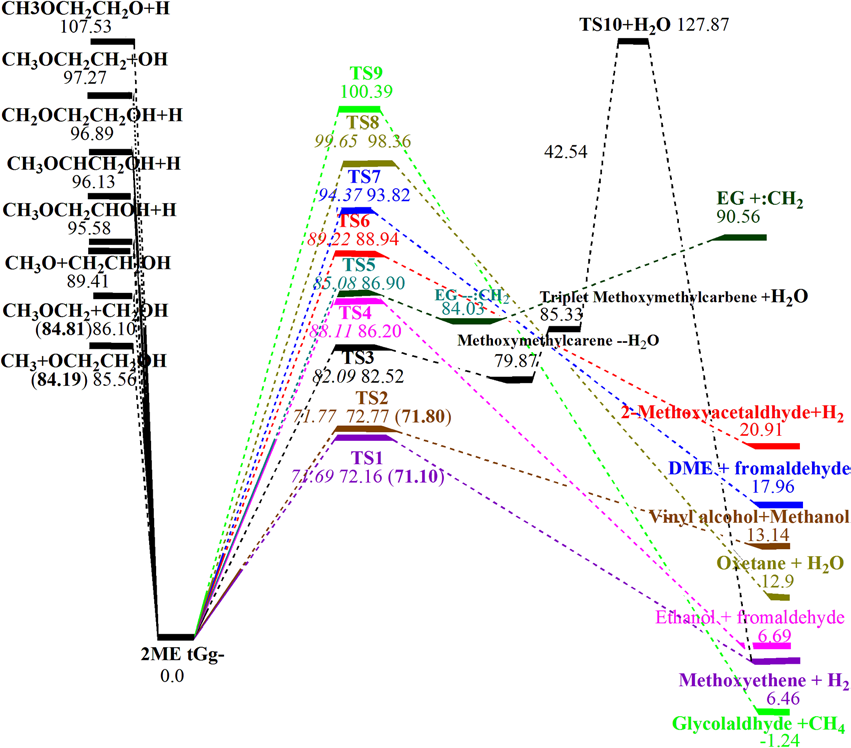
Potential energy diagram of unimolecular decomposition of 2ME (energies in kcal/mol) at G3 *italic*, CBS-QB3 plain, and W1 (**bolded** in parentheses).

**Figure 7:**
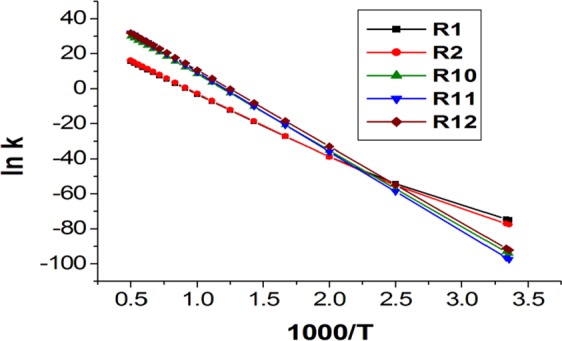
Arrhenius plots for 2ME pyrolysis through decomposition reactions R1, R2, R10, R11, and R12 over the temperature range 298–2000 K.

Detailed optimized structures of products and bonds lengths variations versus IRC of complex fission reactions are given in the SI (Figs 1S–9S), while the potential energy diagrams of 2ME pyrolysis at the G3 and CBS-QB3 methods are shown in Fig. 6 and the results at BMK/6–31+G(d,p) are listed in Table 8S. The barrier heights and reaction energies of the main favorable routes at CBS-QB3 and G3 methods are tested against the W1 method to validate the reliability of their values for the current molecular structure. The results of barrier heights show that the CBS-QB3 and G3 results are in a good agreement with that of W1 with a maximum difference of 1.0 and 0.5 Kcal/mol for CBS-QB3 and G3, respectively. The comparison also indicates a 1.3 Kcal/mol for maximum energy difference of main simple bond fission reactions at CBS-QB3 (see Fig. 6). So, from hereinafter, unless noted otherwise, all results are discussed at the CBS-QB3 level of theory.

### Energies and IRC analysis

Among complex reactions, two reactions (R3 and R5) proceed via three-membered ring transition state, while the rest is passing over the four-membered ring. Almost all reactions are endothermic so that structures of transition states are close to those of products more than reactants according to Hammond postulate^59^. As a result of the high oxygen content in 2ME (42.1% per molecular weight), a theoretical study on 2ME combustion is essential since many oxygenates like ether, alcohols, and carbonyl compounds can be released to the atmosphere during its ignition.

### Formation of ethers compounds

Decomposition of 2ME resembles a platform of many ether categories like methoxyethene, methoxymethyl carbene, dimethyl ether (DME), oxetane, and 2-methoxy acetaldehyde. Methoxyethene formation is the preferable kinetic channel on the potential energy diagram of 2ME decomposition with a barrier height and reaction energy of 72.2 and 6.5 kcal/mol, respectively. The reaction can be accomplished via intra-molecular H-atom abstraction from C_β_ by the alcoholic OH (1,2-water elimination) via the four-membered ring transition state TS1. The selected transition state involves inter-rotational of gauche dihedral angle (OCCO) and anti-gauche dihedral angle (CCOH) to be −108° and 102°, respectively. The IRCs of the methoxyethene formation appear in Fig. 1S. Figure 1S shows a fast rapture for the strong C1-O1 bond (BDE = 97.9 kcal/mol) rather than the weakest C2-H3 bond (BDE = 96.7 kcal/mol). The reason is attributed to the high-frequency factor of the C1-O1 bond compared to that of the C-H bonds (see SI). The broken C2-H3 bond at s = −1 amu^1/2^ bohr is associated with the O1-H3 bond formation, where the two curves cross each other at the transition state (s = −0.2 amu^1/2^ bohr). Formation of the C1-C2 double bond occurs gently during the reaction.

Methoxymethyl carbene is an unstable compound that is obtained by 1,1-water elimination of C_α_ via TS3. The reaction requires a preliminary structure conversion from the most stable conformer tGg- to the tGt structure through multi-steps with final reaction energy of 2.9 kcal/mol. The reaction proceeds via a three-membered ring transition state with barrier energy of 82.5 kcal/mol. The transformation process of 2ME to methoxymethyl carbene is displayed in Fig. 2S. Figure 2S shows a superior rapture for the C1-O1 bond than the C1-H1 one. The disintegration of the C1-O1 bond begins at s = −1.2 amu^1/2^ bohr, while the formation of the O1-H1 bond progresses simultaneously with cracking of the C1-H1 bond. The two curves cross each other at s = −0.5 amu^1/2^ bohr. The slight decrease in values of the C1-O1 bond length after the cracking is a clue for the formation of an intermediate compound with an H-bond linking separated atoms near each other.

Oxetane production has the highest barrier energy value among water elimination reactions from 2ME with a barrier height of 98.3 kcal/mol. The high energy barrier can be attributed to the formation of a highly strained four-membered cyclic product. The reaction proceeds by the alcoholic abstraction of the C_ɤ_ hydrogen (1,4-water elimination) with the four-membered ring transition state TS8. The barrier height and the reaction energy of 98.4 and 12.9 kcal/mol are in line with the work in ref.^60^ where the barrier height and the reaction energy were 96.0 and 15.7 kcal/mol, at the same level of theory, for the same investigated channel of 1,4- dehydration of n-butanol. Table 6 shows a comparison between 2ME and n-butanol with respect to 1,1-, 1,2-, and 1,4- H_2_O elimination reactions. Oxetane is formed over multi-conversion processes as the most stable tGg- converts to tGt then to g-Gt conformer by a rotational barrier of 0.6 kcal/mol and reaction energy of 0.4 kcal/mol relative to tGt conformer (2.7 and 1.5 kcal/mol, respectively in case of n-butanol^60^). Figure 3S illustrates a fast cleavage of the strong C1-O1 bond relative to the weakest C3-H7 bond which occurs at s = 1.5 amu^1/2^ bohr. The formation of the O1-H7 bond starts at s = −0.1 amu^1/2^ bohr. The two curves of C3-H7 and O1-H7 bonds cross each other at s = 0.9 amu^1/2^ bohr, while the formation of the single σ covalent bond C1-C3 occurs gradually during the reaction.

**Table 6:** A comparison of barrier heights and reaction energies (kcal/mol) for 1, 1-; 1, 2-; and 1, 4- water elimination reactions of n-butanol and 2ME in room temperature at CBS-QB3.

path	n-butanol^a^			2ME^b^		
	barrier height	reaction energy	product	barrier height	reaction energy	product
1, 1-	81.2	—	Propyl carbene	82.5	—	Methoxymethyl carbene
1, 2-	67.9	9.3	1-Butene	72.2	6.5	Methoxyethene
1, 4-	95.9	15.7	Cyclobutane	98.3	12.9	Oxetane

^a^Reference ^60^, ^b^Current study.

DME is produced via TS7. The alcoholic H-atom migrates to C_β_ resulting in DME and formaldehyde. The alcoholic H-atom rotates from the gauche dihedral angle of 51° to 0° for facilitating the conversion process. The change of bond lengths for the formation of DME is shown in Fig. 4S. The Figure shows that the weakest C1-C2 bond (BDE = 86.7 kcal/mol) dissociates earlier (at s = −2 amu^1/2^ bohr) than the strong alcoholic O1-H8 bond (BDE = 108.1 kcal/mol) rapture at s = −0.8 amu^1/2^ bohr. The C2-H8 bond is formed at s = 1 amu^1/2^ bohr and the carbonyl C1-O1 bond of formaldehyde is formed smoothly during the reaction. The curves of the O1-H8 and C2-H8 bonds cross each other at the transition state.

2-Methoxyacetaldehyde is a direct result for the 1,2-H_2_ elimination from 2ME. The reaction proceeds via the TS6 with a barrier height and reaction energy of 88.9 and 20.9 kcal/mol, respectively. Figure 5S shows a variation of selected bonds lengths during the formation of 2-methoxy acetaldehyde. It is clear that breaking the weak C1-H1 bond (BDE = 96.2 kcal/mol) occurs first and then the alcoholic O1-H8 bond (BDE = 108.1 kcal/mol), while the carbonyl C1-O1 double bond formation progresses smoothly during the reaction.

### Formation of alcohols and carbonyl compounds

Many alcohols such as methanol, vinyl alcohol, ethanol, glycolaldehyde, and ethylene glycol are released through the combustion of 2ME. Vinyl alcohol production occurs via TS2. It is the 2^nd^ kinetically preferable pathway with a barrier height difference of 0.6 kcal/mol relative to the most stable methoxyethene transition state TS1. The less stable vinyl alcohol (enol) transforms into the most stable acetaldehyde (keto) (TS11) via the 1,3- intramolecular H atom transfer. The reaction barrier is 55.9 kcal/mol and the reaction energy is 11.54 kcal/mol relative to the vinyl alcohol that agrees with our past recorded data^57^ and with alkenol – alkanal conversion using CBS composite methods^61,62^.

According to Fig. 6S, the weakest O2-C2 bond (BDE = 86.7 kcal/mol) is broken first (at s = 2 amu^1/2^ bohr) then the C1-H1 bond (BDE = 96.2 kcal/mol) stretches slowly until rapture at s = 0.9 amu^1/2^ bohr. Fission of the C1-H1 bond and the formation of the alcoholic O2–H8 bond occur at the same time and the two curves cross each other at s = 0.3 amu^1/2^ bohr, while the formation of the enolic double bond C1–C2 occurs step by step during the conversion process.

EG production is the highest endothermic route among all H-atom transfer channels with reaction energy of 90.6 kcal/mol. The reaction proceeds by 1,2- H-atom transfer via TS5 as one of the C_γ_ hydrogen migrates to the O_β_ via a strained three-membered ring transition state. The high recorded reaction energy may be related to the formation of the less stable triplet methylene. The investigation related to the IRC in Fig. 7S indicates a fast breakage of the O2–C3 bond (BDE = 86.1 kcal/mol) at s = 2 amu^1/2^ bohr, while the C3–H6 bond stretches and breaks at s = 1.5 amu^1/2^ bohr with the formation of the O2–H6 bond. The two curves interrupted at s = 0.8 amu^1/2^ bohr. Similar to the methoxymethyl carbene, the variational of the O2–C3 bond length is a clue for the formation of the H-bond which makes the two separated atoms close to each other after the product formation.

Ethanol is produced via TS4 with an energy barrier of 86.2 kcal/mol and reaction energy of 6.7 kcal/mol. The reaction occurs by shifting one of the C_γ_ hydrogens to the C_β_ passing over the etheric oxygen O_β_. Figure 8S reveals that the O2-C2 bond breaks before the C3-H7 bond, which agrees with the bond dissociation values of the two bonds, while the O2-C3 double bond forms slowly during the reaction. Thermodynamically, ethanol formation is preferable than methoxyethene production by 0.2 kcal/mol.

Glycolaldehyde is also a bifunctional compound that has alcohol and aldehyde groups. It is formed through TS9 which is the highest energy barrier among all complex channels (100.4 kcal/mol). However, it is the preferable thermodynamic pathway with reaction energy of −1.2 kcal/mol. Figure 9S in the SI shows the earlier rapture of the least energy O2-C3 bond (BDE = 86.2 kcal/mol), while the C2-H4 bond (BDE = 96.7 kcal/mol) stretches gently till it gets broken at s = −0.6 amu^1/2^ bohr. Formation of the C3-H4 bond occurs at s = 1.5 amu^1/2^ bohr. The two curves of C3-H4 and C2-H4 bonds cross each other at s = 0.7 amu^1/2^ bohr, while the O2-C2 double bond is formed gently during the reaction.

### Rate constant calculation

Figure 7 displays the Arrhenius diagram for the main kinetically favored paths of 2ME decomposition over the temperature range 298–2000 K. For liner relations between ln k vs. 1000/ T for reactions R10, R11, and R12, the activation energy and pre-exponential factor can be derived from the two-parameter Arrhenius equation:

$${{k}^{{\rm{TST}}}}_{(T)}=A\,{{\rm{e}}}^{-{\rm{\Delta }}{E}^{\dagger }/{\rm{R}}T}$$

Taking the Natural Logarithm of the two sides

$$\mathrm{ln}\,{k}^{{\rm{TST}}}(T)=\,\mathrm{ln}\,A-\frac{{\rm{\Delta }}{E}^{\dagger }}{{\rm{R}}T}$$

Plotting ln k^TST^(*T*) versus $\frac{1000}{T}$ shows a straight line with a frequency factor A (s^−1^) = e^Intercept^ and an activation energy ${\rm{\Delta }}{E}^{\dagger }({\rm{cal}}/{\rm{mol}})={\rm{slope}}\times 1.987$.

In Fig. 7, the tunneling correction calculated by Eckert method plays a vital role for the curvature of the relation between ln k vs. 1000/T for R1 and R2 reactions at *T* ≤ 500 K. Therefore, these reactions can fit the three-parameter Arrhenius equation of A, n, and E_a_. Table 7 lists parameters of rate equations of main kinetic paths for 2ME.

**Table 7:** Rate Expressions for the predominant reactions (R1, R2, R10, R11and R12) over the temperature range 298–2000 K at CBS-QB3.

Reaction	A(S^−1^)	n	E_a_ (kcal/mol)
R1	6.30 × 10^−15^	8.07	52.19
R2	2.22 × 10^−10^	6.91	56.10
R10	3.10 × 10^22^	0	86.40
R11	6.31 × 10^23^	0	90.17
R12	2.70 × 10^23^	0	86.41

In the case of three-parameter Arrhenius equation, the following equation is used:

$${{k}^{{\rm{TST}}}}_{(T)}=A{{\rm{T}}}^{n}\,{{\rm{e}}}^{-{\rm{\Delta }}{E}^{\dagger }/{\rm{R}}T}$$

Taking the Natural Logarithm of the two sides gives;

$$\mathrm{ln}\,{k}=\,\mathrm{ln}\,{A}+n\,{\rm{lnT}}-{\rm{\Delta }}{E}^{\ddagger }/\mathrm{RT}$$

$${\rm{At}}\,{\rm{T}}={{\rm{T}}}_{1}\,{\rm{k}}={{\rm{k}}}_{1}.$$

The equation converts to the general form

3 $$\mathrm{ln}\,A+{{\rm{X}}}_{1}n+{Y}_{1}\,{\rm{\Delta }}{E}^{\ddagger }={{\rm{Z}}}_{1}$$

X_1_, Y_1_, and Z_1_ are known values.

By similar at T = T_2_ *k* = *k*_*2*_, and T = T_3_ *k* = *k*_3._

We will get another two equations of the three variables *A*, *n*, and ${\rm{\Delta }}{E}^{\dagger }$.

4 $$\mathrm{ln}\,A+{{\rm{X}}}_{2}n+{{\rm{Y}}}_{2}\,{\rm{\Delta }}{E}^{\ddagger }={{\rm{Z}}}_{2}$$

5 $$\mathrm{ln}\,A+{{\rm{X}}}_{3}n+{{\rm{Y}}}_{3}\,{\rm{\Delta }}{E}^{\ddagger }={{\rm{Z}}}_{3}$$

The algebraic solution of the three Eqs (3), (4) and (5) gives values of *A*, *n*, and ${\rm{\Delta }}{E}^{\dagger }$.

Arrhenius equations for the calculated rate constant (s^−1^) for main channels R1, R2, R10, R11, and R12 in the temperature range 298–2000 K can be summarized as follow:

$${k}_{{\rm{R1}}}({\rm{T}})=6.30\times {10}^{-15}\times {{\rm{T}}}^{8.07}\,\exp \,(-26264/T)$$

$${k}_{{\rm{R2}}}({\rm{T}})=2.22\times {10}^{-10}\times {{\rm{T}}}^{6.91}\,\exp \,(-28233/T)$$

$${k}_{{\rm{R10}}}({\rm{T}})=3.10\times {10}^{22}\,\exp \,(-43485/T)$$

$${k}_{{\rm{R11}}}({\rm{T}})=6.31\times {10}^{23}\,\exp \,(-45379/T)$$

$${k}_{{\rm{R12}}}({\rm{T}})=2.70\times {10}^{23}\,\exp \,(-43487/T)$$

The branching ratio of the dominant paths of 2ME pyrolysis in a temperature range of 298–2000 K is given in Table 8. The results show highly domination of the low energy barrier H atom transfer reaction which leads to the formation of the methoxyethene (R1) with minor contribution of vinyl alcohol formation (R2) at *T* ≤ 400 K. Despite the CTST rates of R1 and R2 reactions are quite equal, the tunneling correction of R1 is higher by a factor of 12.5 at T = 298 K to 3 at 400 K which is the cause of the dominating R1 reaction over R2 at *T* ≤ 400 K. On raising the temperature, a significant contribution from the simple bond scission of C_α_ − C_β_ and C_β_ − O_β_ bonds (R11 and R12) appears, where the higher frequency factors of these channels can overcome the higher activation energies required for them to proceed. Despite raising the temperature, a small contribution is observed for the low activated energy C_α_ − O_β_ bond (E_a_ = 86.4 kcal/mol) compared to the comparable C_α_ − C_β_ bond (E_a_ = 86.4 kcal/mol) and the higher C_β_ − O_β_ bond (E_a_ = 90.2 kcal/mol). This can be attributed to the high-frequency factors of the latter two paths compared to the former one. At *T* ≥ 700 K, the contribution from the C_β_ − O_β_ bond fission increases gradually and starting competing with the fission of the C_α_-C_β_ bond at *T* > 1600 K until they have an almost equal ratio at *T* = 2000 K. In general, the C_α_ − C_β_ bond fission is considered as the most dominated pathway for 2ME especially at *T* ≥ 500 K that matches with records from similar studies on oxygenated compounds^52,57,63,64^. Due to missing of kinetic data of n-butanol at CBS-QB3, the total rate of 2ME pyrolysis is compared with 2-butanol^57^ at the CBS-QB3 at different temperatures. The results show the superiority of 2ME pyrolysis; hence it is a promising biofuel additive.

**Table 8:** Shows branching ratio^a^ (Γ) of main pathways R1, R2, R10, R11, and R12 in the overall reaction of the thermal decomposition of 2ME

Temp/Ratio	Γ_R1_	Γ_R2_	Γ_R10_	Γ_R11_	Γ_R12_	Γ_R13_	Γ_R15_	Γ_R16_	Γ_R17_
298	94.01	5.99	0.00	0.00	0.00	0.00	0.00	0.00	0.00
300	93.69	6.31	0.00	0.00	0.00	0.00	0.00	0.00	0.00
400	51.69	18.04	2.98	0.54	26.74	0.00	0.00	0.00	0.00
500	0.27	0.24	9.04	4.25	86.21	0.00	0.00	0.00	0.00
600	0.01	0.01	8.61	7.60	83.76	0.00	0.00	0.00	0.00
700	0.00	0.00	8.21	11.36	80.43	0.00	0.00	0.00	0.00
800	0.00	0.00	7.93	15.33	76.73	0.01	0.00	0.00	0.00
900	0.00	0.00	7.65	19.19	73.14	0.01	0.00	0.00	0.00
1000	0.00	0.00	7.44	22.88	69.64	0.02	0.00	0.01	0.00
1100	0.00	0.00	7.23	26.35	66.36	0.04	0.00	0.01	0.00
1200	0.00	0.00	7.06	29.58	63.27	0.07	0.01	0.02	0.01
1300	0.00	0.00	6.86	32.52	60.48	0.10	0.01	0.03	0.01
1400	0.00	0.00	6.72	35.23	57.86	0.13	0.01	0.04	0.01
1500	0.00	0.00	6.58	37.69	55.46	0.18	0.02	0.06	0.01
1600	0.00	0.00	6.45	39.97	53.23	0.23	0.02	0.08	0.02
1700	0.00	0.00	6.32	41.99	51.26	0.28	0.03	0.10	0.02
1800	0.00	0.00	6.20	43.86	49.40	0.34	0.04	0.12	0.03
1900	0.00	0.00	6.10	45.50	47.75	0.41	0.05	0.15	0.04
2000	0.00	0.00	6.01	47.15	46.09	0.48	0.05	0.18	0.04

^a^Eckart tunneling correction.

## Conclusions

The current paper presents a detailed theoretical study on 2-methoxyethanol (2ME) pyrolysis utilizing both DFT (BMK/6–31+G(d, p)) and *ab initio* (CBS-QB3 and G3) procedures. A comparison with n-butanol was taken into account due to the structural similarity and absence of experimental data for 2ME. The obtained results can be summarized as follows:

1. All the investigated reactions are endothermic except that form methane and glycolaldehyde.
2. Production of methoxyethene via 1,3-H atom transfer represents the most kinetically favored path in the course of 2ME pyrolysis at room temperature and requires less energy than the weakest C_α_ − C_β_ simple bond fission. Thermodynamically, the preferable channel is methane and glycoladhyde formation.
3. The strength of C_α_ − C_β_ and C_γ_ − O_β_ bonds is very close, which reflects the significant contribution in overall rate constant especially at high temperature.
4. For barrier heights, the results obtained from BMK/6–31+G (d, p) are in poor agreement with G3 and CBS-QB3. The deviation was 0.2–2.5 kcal/mol compared to CBS-QB3 and a significant deviation (5–12 kcal/mol) for channels involving hydrogen atom migration over the etheric group.
5. Comparison between 2ME and n-butanol regarding 1,1-, 1,2-, and 1,4-water elimination shows faster water elimination reactions for n-butanol than that of 2ME.
6. Many oxygenated compounds can be released due to the high oxygen content of 2ME biofuel. Therefore, detailed studies of 2ME oxidation are necessary for suggesting it as an inferior or superior biofuel relative to n-butanol.

## Supplementary information

supporting information

## Data Availability

All data generated through this study are collected in this manuscript and the Supporting Information file.
