# Supplementary material for: Thermochemistry and Kinetics of the Thermal Degradation of 2-Methoxyethanol as Possible Biofuel Additives
Source: Sci Rep. 2019 Mar 14;9:4535. doi: 10.1038/s41598-019-40890-2 (PMC6418115; doi:10.1038/s41598-019-40890-2)
Supplement: Supplementary file 1 — supporting information [file 41598_2019_40890_MOESM1_ESM.docx]

**Thermochemistry and Kinetics of the Thermal Degradation of 2-****Methoxyethanol as Biofuel Additives**

Mohamed A. Abdel-Rahman,^1^ Nessreen Al-Hashimi,^2^ Mohamed F. Shibl,^2^ Kazunari Yoshizawa,^3^ and Ahmed M. El-Nahas^1,3^

^1^Chemistry Department, Faculty of Science, Menoufia University, Shebin El-Kom, Egypt

^2^Department of Chemistry and Earth Sciences, College of Arts and Sciences, Qatar University, P.O. Box 2713, Doha, Qatar

^3^Institute for Materials Chemistry and Engineering and IRCCS, Kyushu University, Fukuoka 819-0395, Japan

**Table of contents:**

Fig. 1S. Change of bond lengths along reaction coordinates for formation methoxyethene and H_2_O.

Fig.2S. Change of bond lengths along reaction coordinates for formation methoxy methyl carbene and H_2_O.

Fig.3S. Change of bond lengths along reaction coordinates for formation oxetane + H_2_O.

Fig.4S. Change of bond lengths along reaction coordinates for formation dimethyl ether and formaldehyde.

Fig.5S. Change of bond lengths along reaction coordinates for formation H_2_ and 2-methoxyaceytaldehyde.

Fig. 6S. Change of bond lengths along reaction coordinates for formation methanol and vinyl alcohol.

Fig. 7S. Change of bond lengths along reaction coordinates for formation ethylene glycol and methylene.

Fig. 8S. Change of bond lengths along reaction coordinates for formation Ethanol and formaldehyde.

Fig. 9S. Change of bond lengths along reaction coordinates for formation glycolaldehyde and methane.

Fig.10S. Correlation between energy barriers (kcal/mol) for complex fission reactions of 2ME.

Fig.11S. Potential energy profiles along MEP of 2ME pyrolysis at BMK/6-31+G (d, p).

Table 1S. Optimized structure and energies of 2ME conformers at CBS-QB3 (right) and BMK/6-31+G (d, p) (left).

Table 2S. Total and relative zero-point corrected energies of 2ME conformers at CBS-QB3.

Table 3S. Total and relative zero-point corrected energies of 2ME conformers at G3.

Table 4S. Total and relative zero-point corrected energies of 2ME conformers at BMK/6-31+G (d, p).

Table 5S. Optimized transition states structures of 2ME pyrolysis at CBS-QB3 (right) and BMK/6-31+G (d, p) (left).

Table 6S. Optimized structures products and radicals of 2ME pyrolysis at CBS-QB3 (right) and BMK/6-31+G (d, p) (left).

Table 7S. Isodesmic equations used in enthalpies of formation calculations.

Table 8 S. Barrier heights and relative energies of different channels of 2ME pyrolysis at BMK/6-31+G(d,p).

Table 9S. Arrhenius coefficient for C_1_-O_1_ and C- H bonds fission reactions.

Table 10S. Rate constants (k, s^-1^) for unimolecular decomposition reactions of 2ME at CBS-QB3.


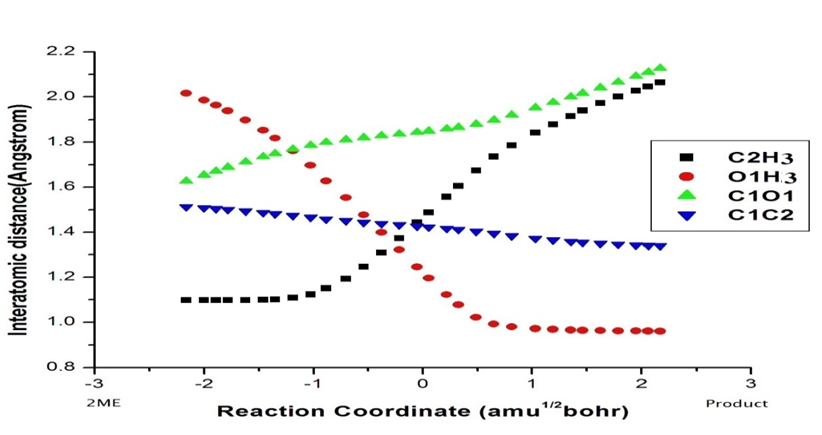


Fig. 1S. Change of bond lengths along reaction coordinates for formation Methoxyethene +H_2_O.


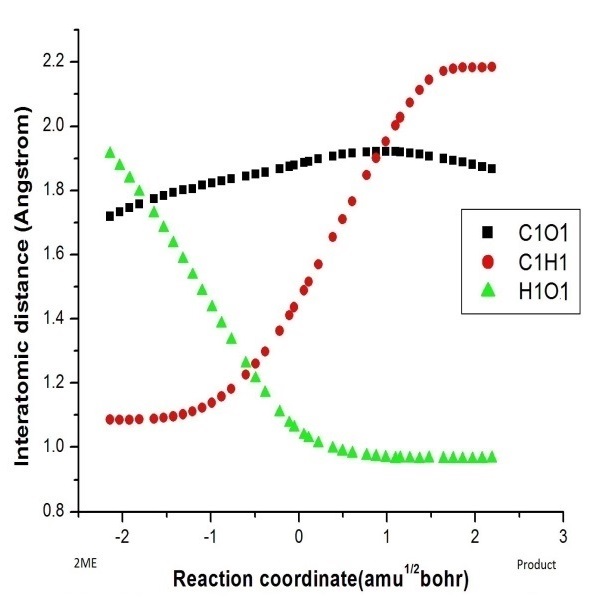


Fig.2S. Change of bond lengths along reaction coordinates for formation methoxy methyl carbene and H_2_O


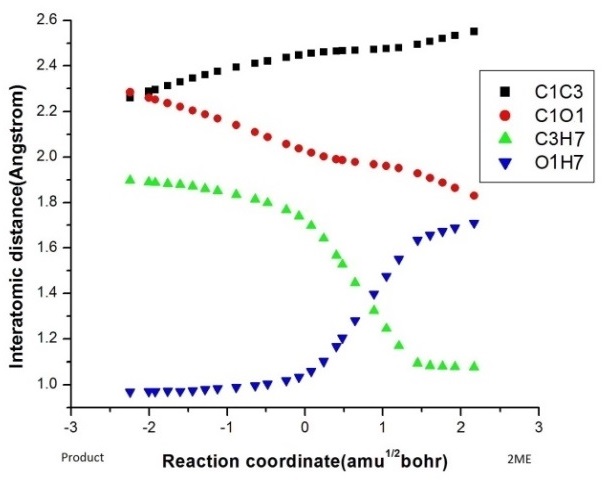


Fig. 3S. Change of bond lengths along reaction coordinates for formation oxetane and H_2_O.


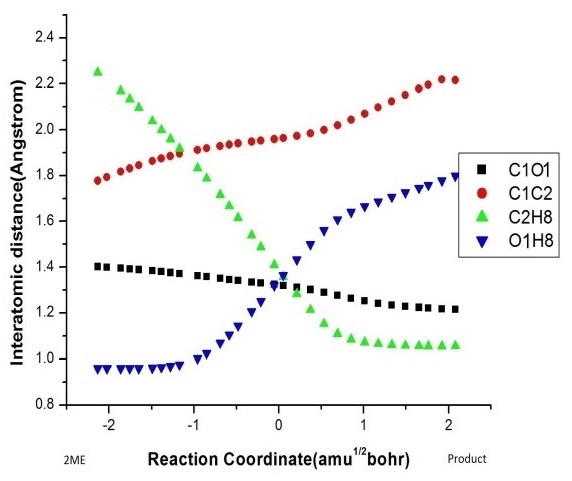


Fig. 4S. Change of bond lengths along reaction coordinates for formation dimethyl ether and formaldehyde.


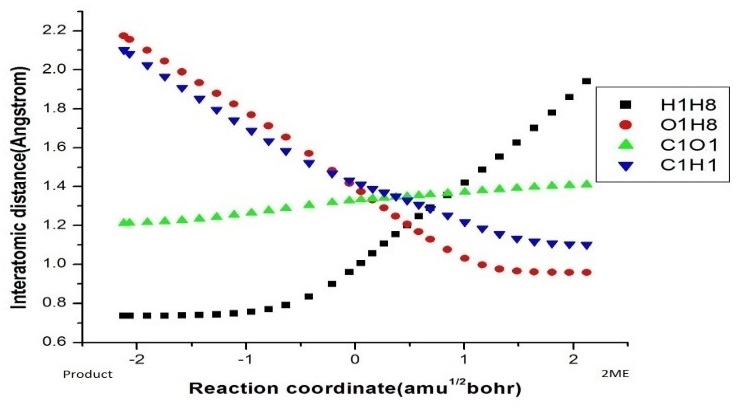


Fig.5S. Change of bond lengths along reaction coordinates for formation H_2_ and 2-methoxyaceytaldehyde.


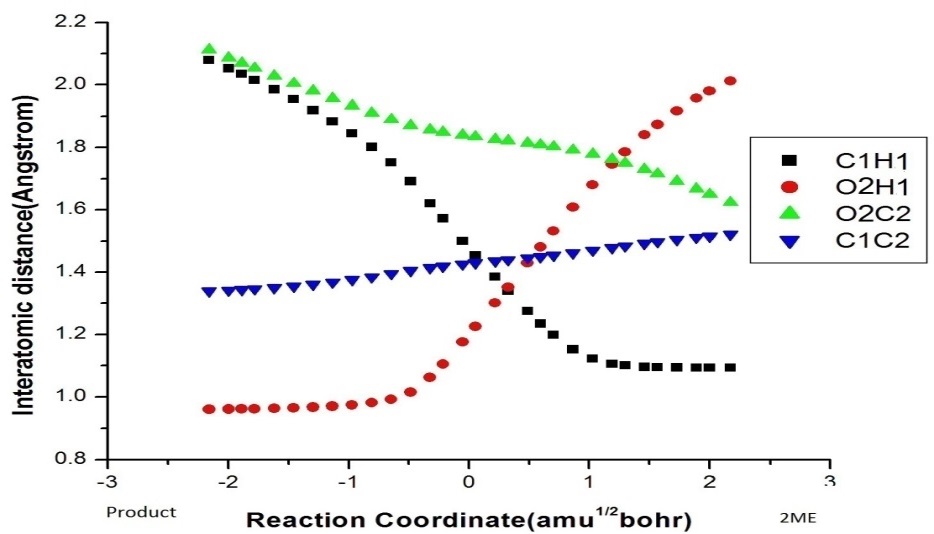


Fig. 6S. Change of bond lengths along reaction coordinates for formation methanol and vinyl alcohol.


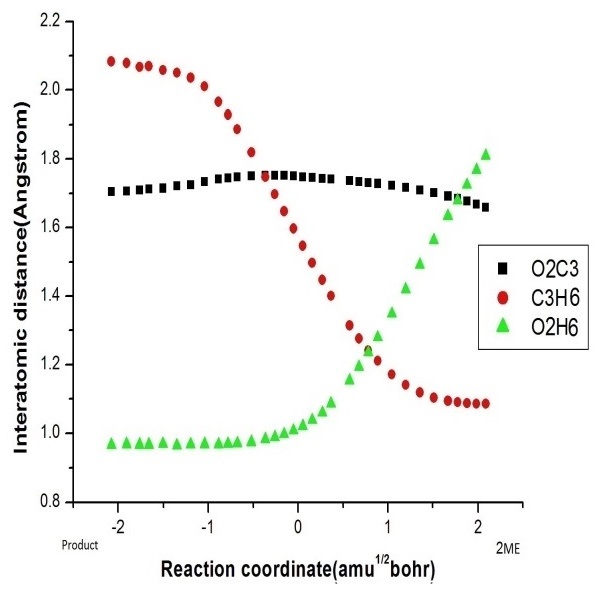


Fig. 7S. Change of bond lengths along reaction coordinates for formation ethylene glycol and methylene


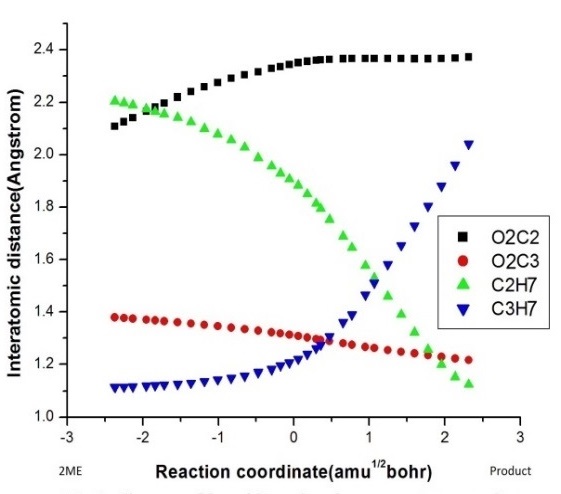


Fig. 8S. Change of bond lengths along reaction coordinates for formation ethanol and formaldehyde.


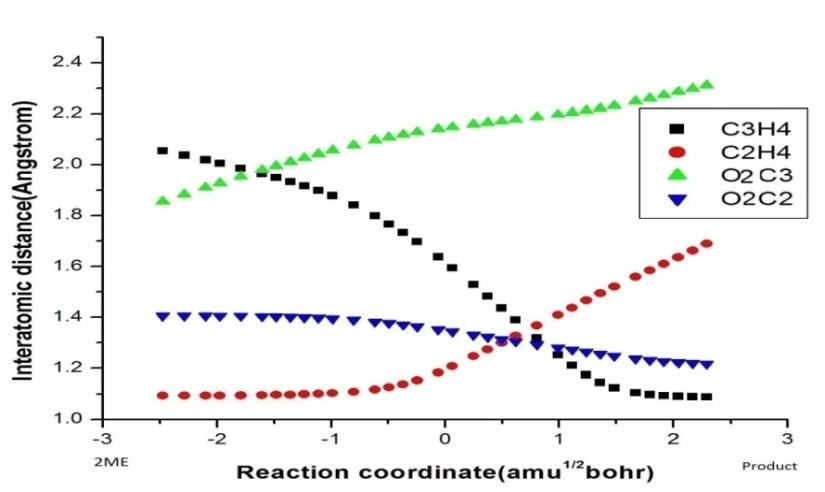


Fig. 9S. Change of bond lengths along reaction coordinates for formation glycolaldhyde and methane.


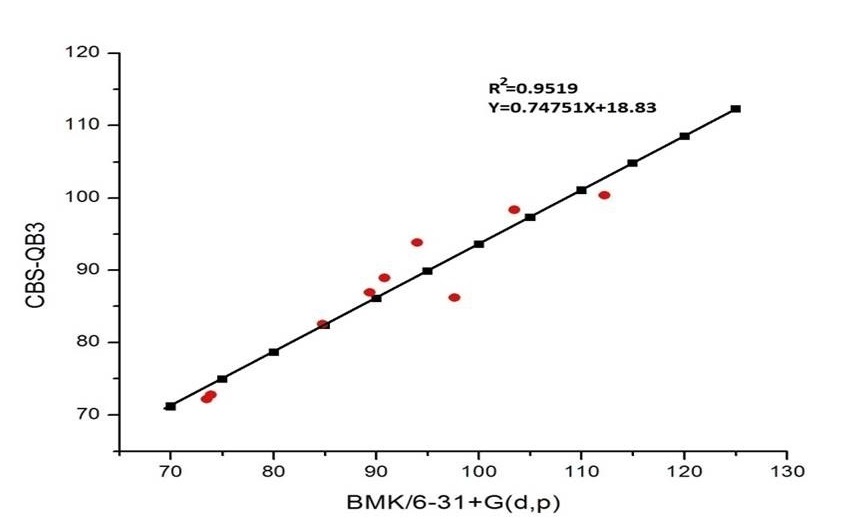


Fig.10S. Correlation between energy barriers (kcal/mol) for complex fission reactions of 2ME.


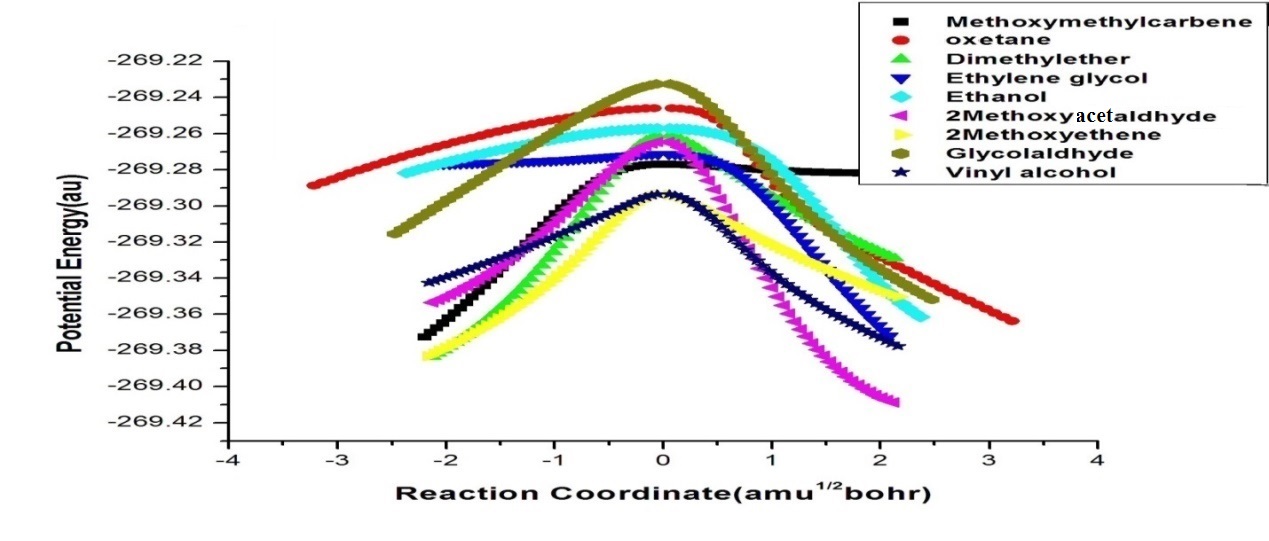


Fig.11S. Potential energy profiles along MEP of 2ME pyrolysis at BMK/6-31+G (d, p).

Table 1S. Optimized structure and energies of 2ME conformers at CBS-QB3 (left) and BMK/6-31+G (d, p)(right).

| 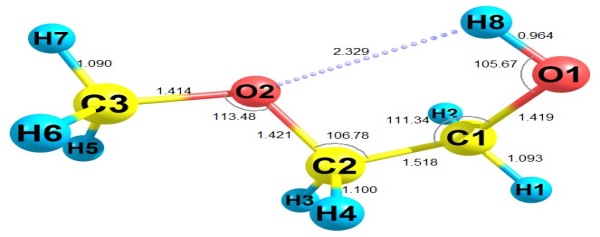  tGg-  6 -1.400078000 0.495495000 -0.218366000  1 -2.069088000 1.239406000 0.220803000  1 -1.451265000 0.591789000 -1.312417000  6 0.025331000 0.752540000 0.234382000  1 0.361754000 1.752955000 -0.079531000  1 0.086787000 0.689809000 1.330798000  8 0.831800000 -0.257820000 -0.356147000  6 2.177564000 -0.228515000 0.076072000  1 2.664846000 0.719841000 -0.190826000  1 2.255255000 -0.367683000 1.163560000  1 2.696864000 -1.045649000 -0.425509000  8 -1.855137000 -0.778858000 0.207421000  1 -1.175360000 -1.404152000 -0.069594000  CBS-QB3 (0 K)= -269.124148  CBS-QB3 Energy= -269.117808  CBS-QB3 Enthalpy=-269.116864  CBS-QB3 Free Energy= -269.153754 | 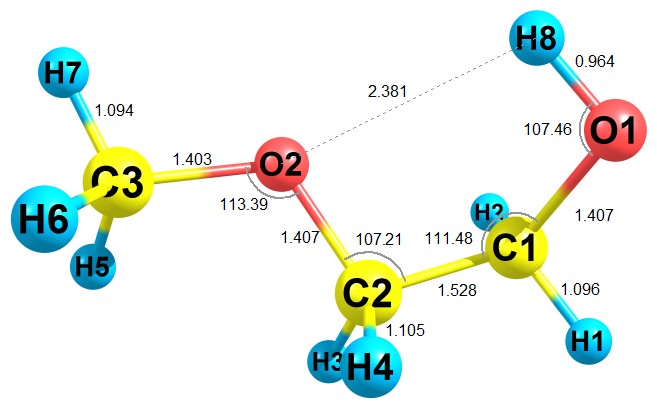  tGg-  6 -1.397804000 0.495173000 -0.222226000  1 -2.056549000 1.254679000 0.214825000  1 -1.445078000 0.580086000 -1.320433000  6 0.040497000 0.738823000 0.232581000  1 0.383160000 1.737872000 -0.092800000  1 0.098627000 0.686649000 1.334319000  8 0.835448000 -0.269527000 -0.342346000  6 2.175958000 -0.225569000 0.070788000  1 2.647386000 0.730856000 -0.211433000  1 2.259042000 -0.352273000 1.163211000  1 2.702296000 -1.044687000 -0.428809000  8 -1.869069000 -0.758234000 0.209033000  1 -1.231815000 -1.421661000 -0.079233000  Sum of electronic and zero-point Energies= -269.304142  Sum of electronic and thermal Energies= -269.297948  Sum of electronic and thermal Enthalpies= -269.297003  Sum of electronic and thermal Free Energies= -269.333546 |
| --- | --- |
| 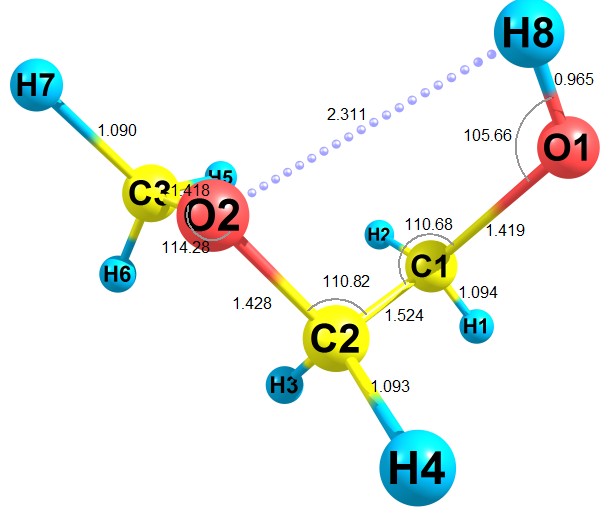  gGg-  6 0.019063000 0.909700000 -0.272578000  1 -0.479052000 1.726975000 0.268228000  1 0.405198000 1.297239000 -1.218290000  6 1.168450000 0.326377000 0.540005000  1 1.924769000 1.095404000 0.719808000  1 0.806717000 -0.017075000 1.521074000  8 1.797783000 -0.729194000 -0.168460000  1 1.080497000 -1.263955000 -0.530472000  8 -0.918037000 -0.107236000 -0.628641000  6 -1.898282000 -0.369548000 0.362111000  1 -1.461728000 -0.722336000 1.305384000  1 -2.500956000 0.525544000 0.568328000  1 -2.548798000 -1.149531000 -0.034478000  CBS-QB3 (0 K)= -269.121671  CBS-QB3 Energy= -269.115309  CBS-QB3 Enthalpy= -269.114365  CBS-QB3 Free Energy= -269.151488 | 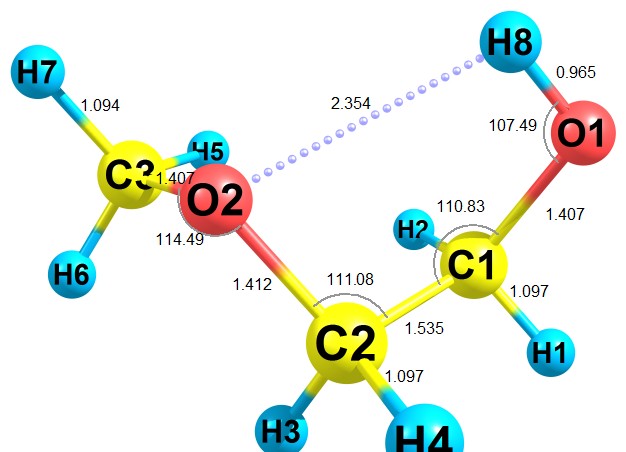  gGg-  6 0.004829000 0.921719000 -0.243219000  1 -0.486804000 1.716700000 0.343852000  1 0.397440000 1.354910000 -1.171187000  6 1.159603000 0.296349000 0.551738000  1 1.912930000 1.062962000 0.771749000  1 0.793393000 -0.099350000 1.514852000  8 1.792992000 -0.712929000 -0.196203000  1 1.101978000 -1.245988000 -0.607631000  8 -0.931126000 -0.059737000 -0.636438000  6 -1.868626000 -0.402220000 0.355226000  1 -1.391132000 -0.829922000 1.251223000  1 -2.460507000 0.478043000 0.656156000  1 -2.537064000 -1.151109000 -0.080352000  Sum of electronic and zero-point Energies= -269.301583  Sum of electronic and thermal Energies= -269.295366  Sum of electronic and thermal Enthalpies= -269.294422  Sum of electronic and thermal Free Energies=-269.331216 |
| 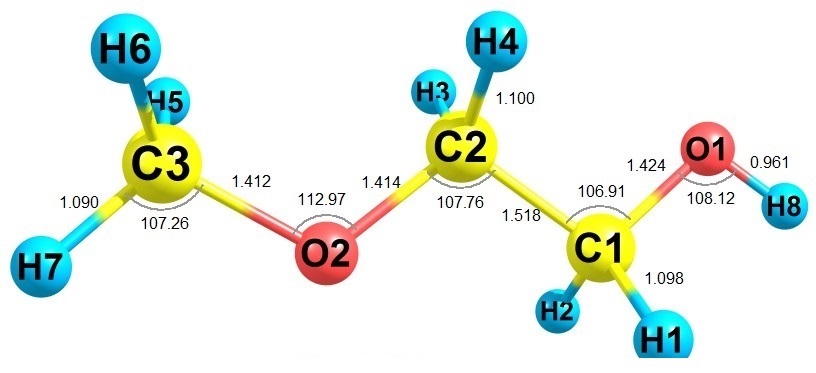 tTt  6 -0.022798000 -0.359999000 0.000000000  1 -0.023746000 -1.008283000 -0.888843000  1 -0.023750000 -1.008282000 0.888843000  6 1.228043000 0.499447000 0.000001000  1 1.218394000 1.143281000 -0.889267000  1 1.218396000 1.143277000 0.889272000  8 2.341090000 -0.388943000 -0.000002000  1 3.144120000 0.138266000 0.000010000  8 -1.141084000 0.505854000 -0.000003000  6 -2.372821000 -0.184744000 0.000002000  1 -2.481845000 -0.818579000 -0.892062000  1 -2.481842000 -0.818572000 0.892071000  1 -3.164311000 0.565372000 -0.000001000  CBS-QB3 (0 K)= -269.120403  CBS-QB3 Energy= -269.113642  CBS-QB3 Enthalpy= -269.112698  CBS-QB3 Free Energy= -269.150540 | 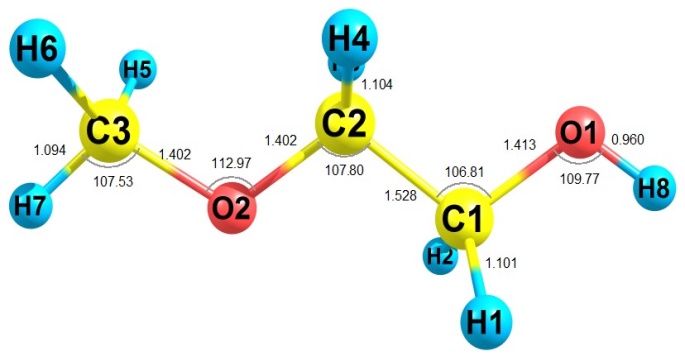  tTt  6 -0.031672000 -0.360030000 0.000001000  1 -0.035481000 -1.008985000 -0.893556000  1 -0.035483000 -1.008982000 0.893559000  6 1.230895000 0.500617000 0.000001000  1 1.226747000 1.143426000 -0.893779000  1 1.226750000 1.143424000 0.893782000  8 2.330170000 -0.386802000 -0.000002000  1 3.150679000 0.112391000 0.000009000  8 -1.137869000 0.501691000 -0.000002000  6 -2.362577000 -0.180381000 0.000000000  1 -2.466599000 -0.816300000 -0.895718000  1 -2.466598000 -0.816297000 0.895722000  1 -3.158298000 0.570977000 0.000000000  Sum of electronic and zero-point Energies=-269.300626  Sum of electronic and thermal Energies= -269.293994  Sum of electronic and thermal Enthalpies= -269.293050  Sum of electronic and thermal Free Energies= -269.330591 |
| 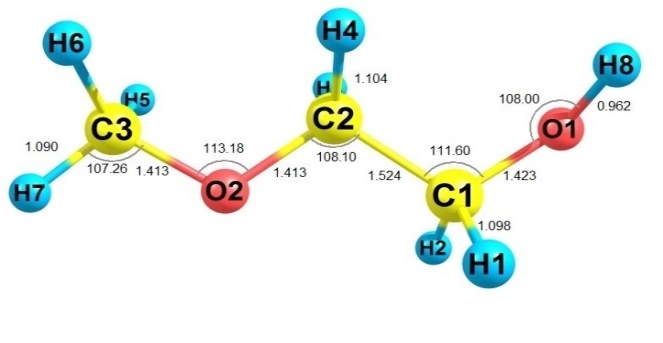  tTg  6 -0.020380000 -0.353824000 0.029080000  1 -0.013162000 -1.062602000 -0.817330000  1 -0.031102000 -0.945860000 0.956237000  6 1.233952000 0.511300000 -0.007917000  1 1.225044000 1.126711000 -0.916617000  1 1.234279000 1.184895000 0.850401000  8 2.415378000 -0.274528000 0.094753000  1 2.507846000 -0.780036000 -0.718531000  8 -1.144458000 0.499267000 -0.047850000  6 -2.373913000 -0.194050000 0.011132000  1 -2.480147000 -0.901447000 -0.824321000  1 -2.479718000 -0.750212000 0.953611000  1 -3.168360000 0.550092000 -0.052447000  CBS-QB3 (0 K)= -269.120088  CBS-QB3 Energy= -269.113432  CBS-QB3 Enthalpy= -269.112488  CBS-QB3 Free Energy= -269.150071 | 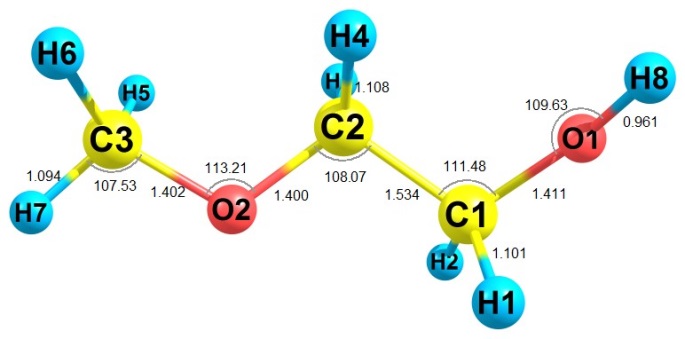  tTg  6 -0.029755000 -0.354635000 0.040439000  1 -0.028244000 -1.074050000 -0.802156000  1 -0.042681000 -0.936836000 0.978908000  6 1.236184000 0.510899000 -0.008801000  1 1.226400000 1.119949000 -0.925918000  1 1.243040000 1.192028000 0.848780000  8 2.403450000 -0.275186000 0.089713000  1 2.527933000 -0.771895000 -0.724057000  8 -1.140066000 0.494694000 -0.042895000  6 -2.363364000 -0.189034000 0.003018000  1 -2.463126000 -0.886738000 -0.846317000  1 -2.467854000 -0.760142000 0.941145000  1 -3.160924000 0.558237000 -0.052866000  Sum of electronic and zero-point Energies= -269.300224  Sum of electronic and thermal Energies= -269.293638  Sum of electronic and thermal Enthalpies= -269.292694  Sum of electronic and thermal Free Energies=-269.330119 |
| 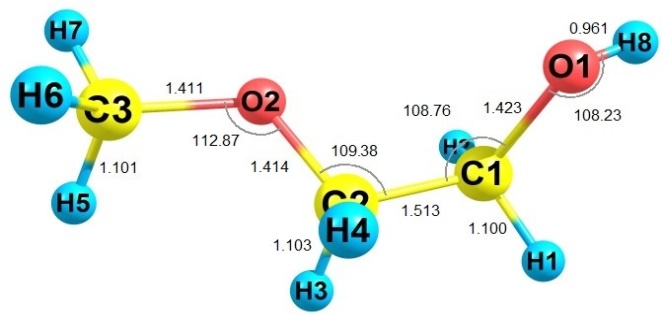  tGt  6 0.059203000 0.640658000 0.285177000  1 0.391361000 1.684814000 0.162478000  1 0.064380000 0.406864000 1.360621000  6 -1.351758000 0.499088000 -0.241471000  1 -1.940917000 1.362280000 0.103171000  1 -1.314335000 0.521200000 -1.338396000  8 -1.900094000 -0.721629000 0.241585000  1 -2.702921000 -0.906884000 -0.252594000  8 0.914224000 -0.234702000 -0.422808000  6 2.230253000 -0.246365000 0.085877000  1 2.698999000 0.748071000 0.029229000  1 2.257481000 -0.583646000 1.132220000  1 2.806719000 -0.942336000 -0.524444000  CBS-QB3 (0 K)= -269.119980  CBS-QB3 Energy= -269.113253  CBS-QB3 Enthalpy= -269.112309  CBS-QB3 Free Energy= -269.150175 | 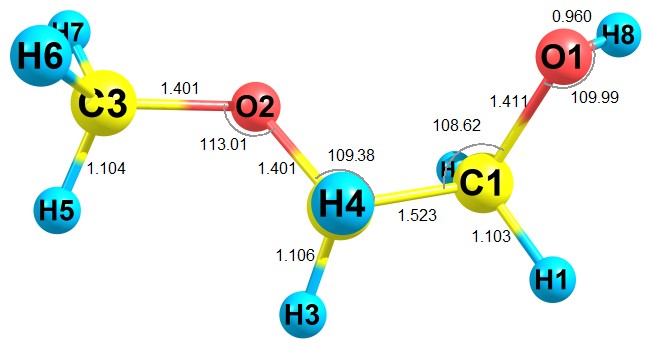  tGt  6 0.067920000 0.641153000 0.282843000  1 0.400462000 1.688903000 0.158792000  1 0.075897000 0.400681000 1.361158000  6 -1.355189000 0.497007000 -0.240465000  1 -1.948654000 1.358513000 0.108869000  1 -1.323856000 0.511569000 -1.340546000  8 -1.894182000 -0.712884000 0.246950000  1 -2.651887000 -0.971755000 -0.282962000  8 0.912778000 -0.224723000 -0.423444000  6 2.217883000 -0.252139000 0.085138000  1 2.688521000 0.746029000 0.041997000  1 2.231630000 -0.601880000 1.131518000  1 2.795434000 -0.947335000 -0.531977000  Sum of electronic and zero-point Energies= -269.299364  Sum of electronic and thermal Energies= -269.292690  Sum of electronic and thermal Enthalpies= -269.291746  Sum of electronic and thermal Free Energies= -269.329559 |
| 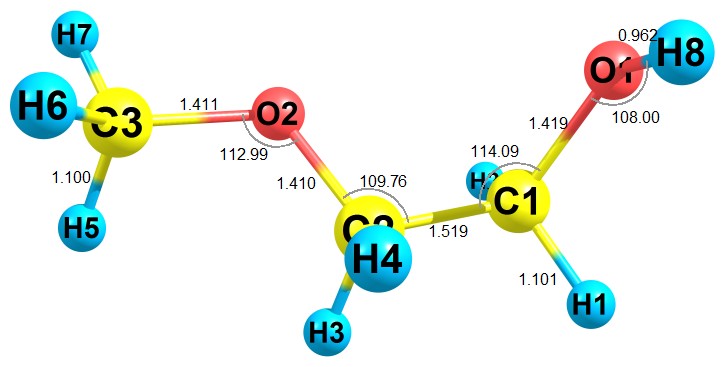  tGg  6 0.057878000 0.649056000 0.277138000  1 0.400973000 1.683542000 0.104284000  1 0.081298000 0.478486000 1.368817000  6 -1.364786000 0.506713000 -0.236748000  1 -1.958189000 1.345229000 0.159482000  1 -1.358944000 0.584251000 -1.325896000  8 -1.965847000 -0.743639000 0.061003000  1 -1.952455000 -0.860097000 1.016136000  8 0.897471000 -0.272403000 -0.382531000  6 2.232292000 -0.226894000 0.074017000  1 2.685501000 0.762594000 -0.088345000  1 2.303228000 -0.469172000 1.144885000  1 2.793291000 -0.969742000 -0.493589000  CBS-QB3 (0 K)= -269.119308  CBS-QB3 Energy= -269.112701  CBS-QB3 Enthalpy= -269.111757  CBS-QB3 Free Energy= -269.149334 | 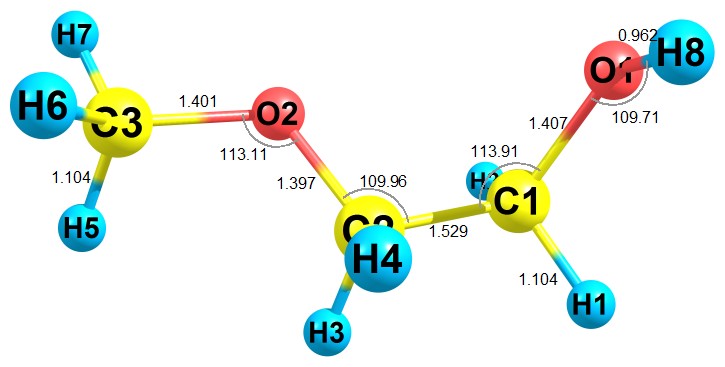  tGg  6 0.065848000 0.637347000 0.283004000  1 0.409763000 1.677516000 0.122901000  1 0.088356000 0.449042000 1.375981000  6 -1.366570000 0.505496000 -0.235603000  1 -1.958288000 1.343910000 0.170913000  1 -1.360065000 0.586941000 -1.328380000  8 -1.967638000 -0.732597000 0.057515000  1 -1.928905000 -0.895866000 1.004723000  8 0.899064000 -0.265721000 -0.382461000  6 2.225048000 -0.226398000 0.069575000  1 2.669504000 0.773975000 -0.074988000  1 2.290601000 -0.488795000 1.139826000  1 2.791669000 -0.958847000 -0.513265000  Sum of electronic and zero-point Energies= -269.298751  Sum of electronic and thermal Energies= -269.292203  Sum of electronic and thermal Enthalpies= -269.291259  Sum of electronic and thermal Free Energies=-269.328708 |
| 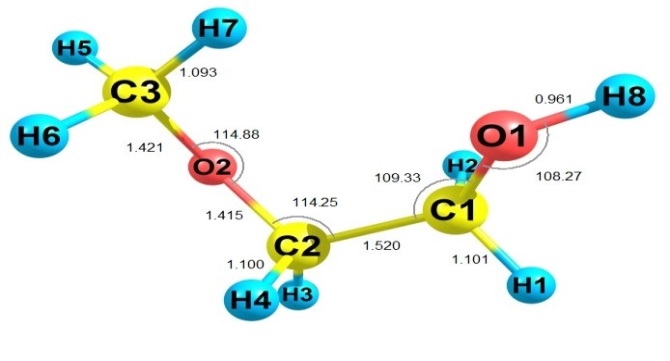 g-Gt  6 0.011111000 0.956103000 0.344721000  1 -0.002076000 2.046743000 0.255118000  1 0.075056000 0.701195000 1.413026000  6 -1.293478000 0.417300000 -0.219564000  1 -2.123010000 1.014388000 0.189738000  1 -1.279397000 0.544396000 -1.310116000  8 -1.432166000 -0.957350000 0.140500000  1 -2.194061000 -1.315138000 -0.324029000  8 1.166875000 0.523139000 -0.348041000  6 1.655722000 -0.752847000 0.043157000  1 1.867418000 -0.779800000 1.121885000  1 0.949977000 -1.553436000 -0.193785000  1 2.588290000 -0.907991000 -0.501391000  CBS-QB3 (0 K)= -269.119173  CBS-QB3 Energy= -269.112607  CBS-QB3 Enthalpy= -269.111663  CBS-QB3 Free Energy= -269.149064 | 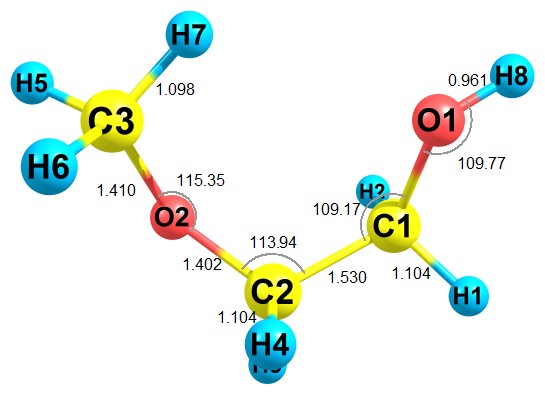  g-Gt  6 0.021204000 0.953151000 0.342225000  1 0.008749000 2.047642000 0.251753000  1 0.091579000 0.691675000 1.412816000  6 -1.298553000 0.415056000 -0.214903000  1 -2.124418000 1.007414000 0.215009000  1 -1.297545000 0.548978000 -1.308089000  8 -1.426864000 -0.951485000 0.131770000  1 -2.219368000 -1.316086000 -0.271353000  8 1.155389000 0.511228000 -0.354308000  6 1.658548000 -0.743751000 0.045637000  1 1.889298000 -0.745793000 1.124471000  1 0.951373000 -1.556023000 -0.166042000  1 2.584935000 -0.902487000 -0.516010000  Sum of electronic and zero-point Energies= -269.298563  Sum of electronic and thermal Energies= -269.292243  Sum of electronic and thermal Enthalpies= -269.291298  Sum of electronic and thermal Free Energies= -269.328226 |
| 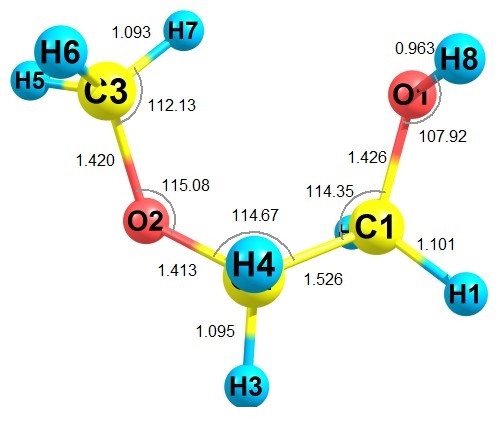  g-Gg  6 0.004194000 0.960862000 0.351914000  1 -0.015831000 2.052275000 0.264173000  1 0.055889000 0.716361000 1.427566000  6 -1.288399000 0.408814000 -0.242868000  1 -2.135025000 0.995698000 0.145782000  1 -1.261259000 0.535963000 -1.327034000  8 -1.489331000 -0.985784000 -0.021028000  1 -1.583582000 -1.124429000 0.926905000  8 1.182594000 0.543403000 -0.307495000  6 1.631852000 -0.760989000 0.030538000  1 1.765554000 -0.860965000 1.118527000  1 0.941333000 -1.536216000 -0.312443000  1 2.600931000 -0.891761000 -0.452798000  CBS-QB3 (0 K)= -269.118271  CBS-QB3 Energy= -269.111808  CBS-QB3 Enthalpy= -269.110864  CBS-QB3 Free Energy= -269.148012 | 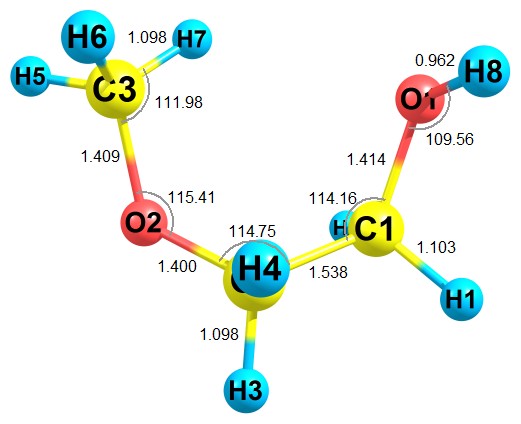  g-Gg  6 0.030667000 0.968608000 0.348508000  1 0.020326000 2.061960000 0.244589000  1 0.033988000 0.729698000 1.431107000  6 -1.249310000 0.408044000 -0.293621000  1 -2.100331000 1.046209000 -0.000066000  1 -1.149602000 0.454421000 -1.384464000  8 -1.507485000 -0.947371000 0.017686000  1 -1.738204000 -1.025786000 0.948062000  8 1.222444000 0.538647000 -0.247955000  6 1.577180000 -0.801138000 0.007068000  1 1.577167000 -1.005611000 1.092148000  1 0.893339000 -1.507377000 -0.481591000  1 2.592426000 -0.936806000 -0.379360000  Sum of electronic and zero-point Energies= -269.298112  Sum of electronic and thermal Energies=-269.291780  Sum of electronic and thermal Enthalpies= -269.290836  Sum of electronic and thermal Free Energies= -269.327734 |
| 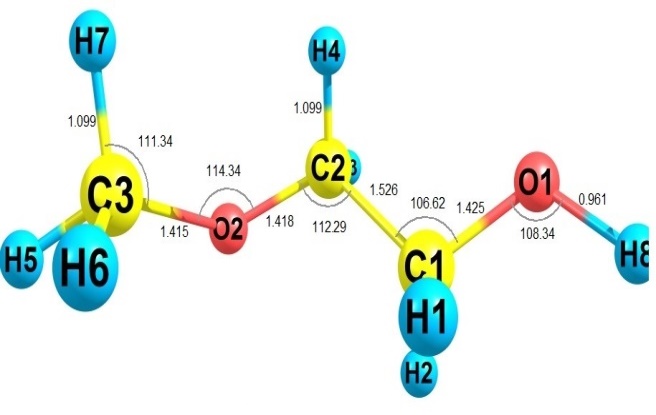  gTt  6 -0.013926000 -0.643467000 0.195365000  1 0.036813000 -0.533340000 1.287302000  1 -0.346727000 -1.660150000 -0.025255000  6 -1.034656000 0.341340000 -0.367594000  1 -0.710135000 1.374274000 -0.179584000  1 -1.102132000 0.197608000 -1.453949000  8 -2.271119000 0.063323000 0.283580000  1 -2.944726000 0.638613000 -0.088390000  8 1.265081000 -0.490023000 -0.397541000  6 2.078218000 0.493149000 0.215497000  1 1.652388000 1.502605000 0.139708000  1 2.248595000 0.265959000 1.277513000  1 3.036410000 0.481899000 -0.305264000  CBS-QB3 (0 K)= -269.118065  CBS-QB3 Energy= -269.111296  CBS-QB3 Enthalpy= -269.110352  CBS-QB3 Free Energy= -269.148457 | 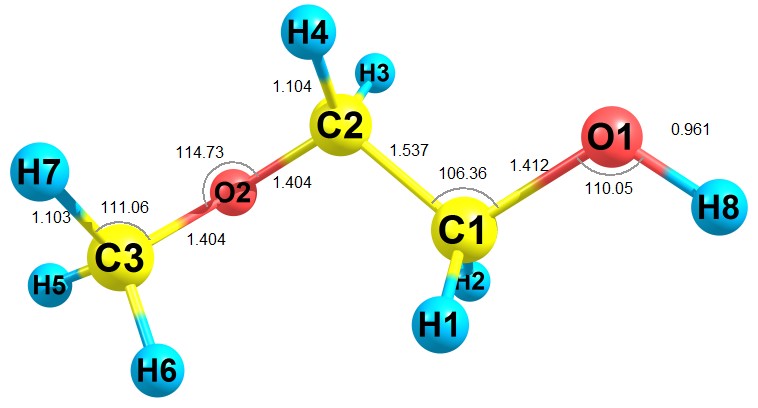  gTt  6 0.008090000 0.677468000 0.178878000  1 -0.023436000 0.634669000 1.281366000  1 0.357762000 1.673613000 -0.115201000  6 1.009454000 -0.367834000 -0.338332000  1 0.683452000 -1.382579000 -0.058670000  1 1.051256000 -0.306011000 -1.437067000  8 2.253076000 -0.054264000 0.253271000  1 2.929420000 -0.659403000 -0.061535000  8 -1.274480000 0.515369000 -0.368751000  6 -2.030647000 -0.520980000 0.202879000  1 -1.628977000 -1.517106000 -0.042471000  1 -2.075554000 -0.419904000 1.300544000  1 -3.044070000 -0.444037000 -0.203678000  Sum of electronic and zero-point Energies= -269.297933  Sum of electronic and thermal Energies= -269.291283  Sum of electronic and thermal Enthalpies= -269.290339  Sum of electronic and thermal Free Energies= -269.328254 |
| 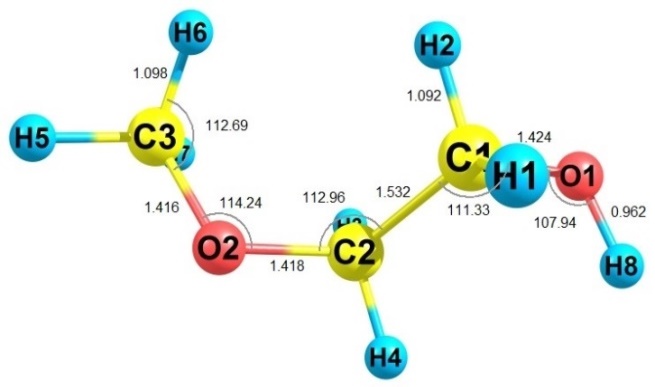  gTg-  6 0.020703000 0.629563000 0.221439000  1 -0.028269000 0.499669000 1.311762000  1 0.365022000 1.650338000 0.022700000  6 1.028837000 -0.364454000 -0.363810000  1 0.716986000 -1.391415000 -0.163542000  1 1.073886000 -0.235653000 -1.453168000  8 2.308116000 -0.223843000 0.245360000  1 2.664784000 0.633015000 -0.008754000  8 -1.264363000 0.526424000 -0.369032000  6 -2.081584000 -0.486418000 0.190139000  1 -1.675411000 -1.493888000 0.031455000  1 -2.224741000 -0.333489000 1.269224000  1 -3.050013000 -0.421374000 -0.306912000  CBS-QB3 (0 K)= -269.117855  CBS-QB3 Energy= -269.111205  CBS-QB3 Enthalpy= -269.110260  CBS-QB3 Free Energy= -269.148015 | 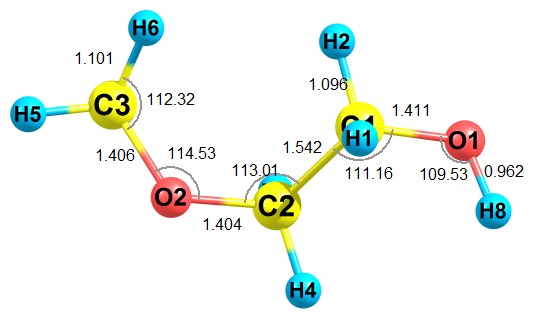  gTg-  6 0.015039000 0.665216000 0.207716000  1 -0.021359000 0.607044000 1.309819000  1 0.376354000 1.665894000 -0.067205000  6 1.001310000 -0.395000000 -0.323394000  1 0.688071000 -1.400650000 -0.021145000  1 1.013652000 -0.360320000 -1.424328000  8 2.289125000 -0.212759000 0.224549000  1 2.667002000 0.606790000 -0.107870000  8 -1.269082000 0.546898000 -0.347045000  6 -2.035397000 -0.509260000 0.175383000  1 -1.644464000 -1.495618000 -0.119797000  1 -2.076803000 -0.461113000 1.276663000  1 -3.048502000 -0.400877000 -0.224397000  Sum of electronic and zero-point Energies= -269.297635  Sum of electronic and thermal Energies= -269.291101  Sum of electronic and thermal Enthalpies= -269.290157  Sum of electronic and thermal Free Energies= -269.327663 |
| 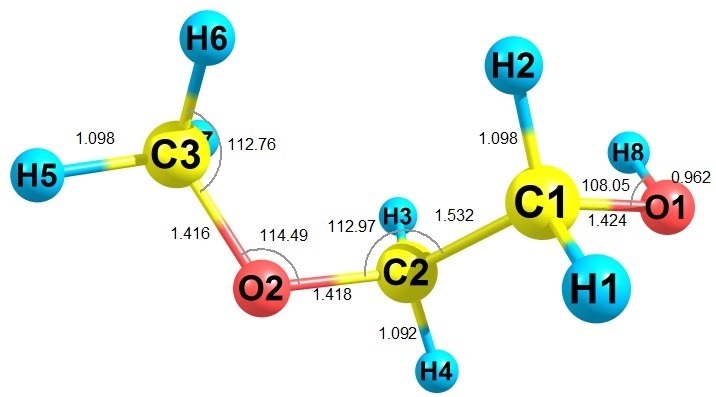  gTg  6 0.018937000 0.632813000 0.229604000  1 -0.030253000 0.463723000 1.318081000  1 0.357860000 1.658534000 0.068113000  6 1.035420000 -0.324119000 -0.401042000  1 0.715331000 -1.367937000 -0.281852000  1 1.103617000 -0.118725000 -1.470583000  8 2.339910000 -0.119102000 0.132427000  1 2.331408000 -0.393345000 1.054671000  8 -1.271291000 0.529491000 -0.349233000  6 -2.072972000 -0.507547000 0.185370000  1 -1.657465000 -1.506000000 -0.003808000  1 -2.212245000 -0.386250000 1.269493000  1 -3.045520000 -0.439989000 -0.303258000  CBS-QB3 (0 K)= -269.117541  CBS-QB3 Energy= -269.110857  CBS-QB3 Enthalpy= -269.109913  CBS-QB3 Free Energy= -269.147770 | 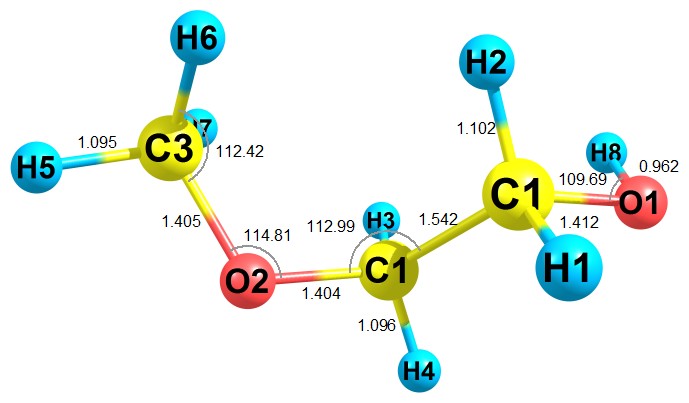  gTg  6 0.011819000 0.668829000 0.207826000  1 -0.018386000 0.565132000 1.309985000  1 0.356893000 1.682385000 -0.026898000  6 1.015599000 -0.341481000 -0.383714000  1 0.685706000 -1.376122000 -0.198699000  1 1.072850000 -0.196081000 -1.467985000  8 2.315048000 -0.122644000 0.122417000  1 2.344777000 -0.362327000 1.053275000  8 -1.277918000 0.539938000 -0.331286000  6 -2.027759000 -0.530314000 0.183794000  1 -1.617892000 -1.509780000 -0.108985000  1 -2.078827000 -0.483527000 1.285212000  1 -3.040114000 -0.440244000 -0.222392000  Sum of electronic and zero-point Energies= -269.297404  Sum of electronic and thermal Energies= -269.290808  Sum of electronic and thermal Enthalpies= -269.289864  Sum of electronic and thermal Free Energies= -269.327575 |
| 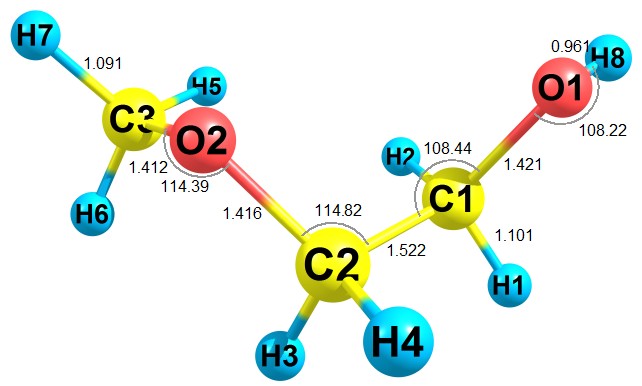  gGt  6 -0.026581000 0.896085000 -0.089294000  1 -0.480032000 1.596201000 0.630930000  1 0.387971000 1.475878000 -0.917421000  6 1.115212000 0.158379000 0.594688000  1 1.739568000 0.908289000 1.105363000  1 0.723215000 -0.523576000 1.362630000  8 1.853262000 -0.545133000 -0.395700000  1 2.520882000 -1.075650000 0.047402000  8 -1.017292000 0.060327000 -0.658903000  6 -1.926296000 -0.485673000 0.273876000  1 -1.447713000 -1.180597000 0.977217000  1 -2.432016000 0.302149000 0.852830000  1 -2.673642000 -1.036982000 -0.297738000  CBS-QB3 (0 K)= -269.117548  CBS-QB3 Energy= -269.110831  CBS-QB3 Enthalpy= -269.109887  CBS-QB3 Free Energy=-269.148368 | 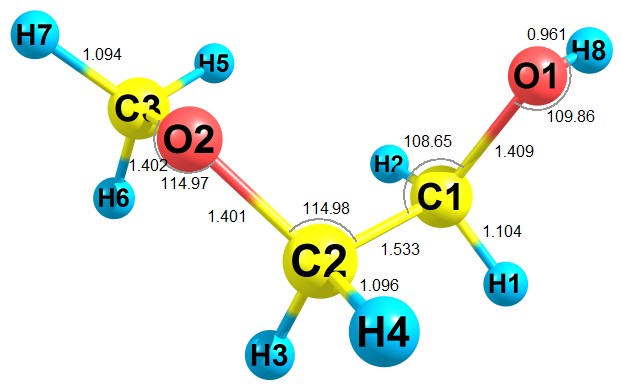  gGt  6 -0.037888000 0.948924000 -0.039543000  1 -0.467072000 1.602305000 0.744178000  1 0.372696000 1.578346000 -0.837710000  6 1.107450000 0.145050000 0.585705000  1 1.786595000 0.865793000 1.073269000  1 0.720219000 -0.531436000 1.365530000  8 1.762644000 -0.573298000 -0.433770000  1 2.460872000 -1.113901000 -0.055098000  8 -1.044727000 0.178624000 -0.635790000  6 -1.818483000 -0.576533000 0.256139000  1 -1.248680000 -1.406378000 0.702987000  1 -2.223905000 0.053614000 1.067759000  1 -2.650534000 -0.995594000 -0.318247000  Sum of electronic and zero-point Energies= -269.296545  Sum of electronic and thermal Energies= -269.290137  Sum of electronic and thermal Enthalpies= -269.289193  Sum of electronic and thermal Free Energies= -269.326477 |

Table 2S. Total and relative zero-point corrected energies of 2ME conformers at CBS-QB3.

| Conformer | E_0_ (hartree) | Relative energy (hartree) | relative energy (kcal/mol) |
| --- | --- | --- | --- |
| tGg- | -269.117808 | 0.0 | 0.0 |
| gGg- | -269.115309 | 0.002499 | 1.57 |
| tTt | -269.113642 | 0.004166 | 2.61 |
| tTg | -269.113432 | 0.004376 | 2.75 |
| tGt | -269.113253 | 0.004555 | 2.86 |
| tGg | -269.112701 | 0.005107 | 3.20 |
| g-Gt | -269.112607 | 0.005201 | 3.26 |
| g-Gg | -269.111808 | 0.006 | 3.76 |
| gTt | -269.111296 | 0.006512 | 4.09 |
| gTg- | -269.111205 | 0.006603 | 4.14 |
| gTg | -269.110857 | 0.006951 | 4.36 |
| gGt | -269.110831 | 0.006977 | 4.38 |

Table 3S. Total and relative zero-point corrected energies of 2ME conformers at G3.

| Conformer | E_0_ (hartree) | Relative energy  (hartree) | Relative energy  (kcal/mol) |
| --- | --- | --- | --- |
| tGg- | -269.338606 | 0.0 | 0.0 |
| gGg- | -269.336183 | 0.002423 | 1.52 |
| tTt | -269.334513 | 0.004093 | 2.57 |
| tTg | -269.334399 | 0.004207 | 2.64 |
| tGt | -269.333961 | 0.004645 | 2.91 |
| tGg | -269.333464 | 0.005142 | 3.23 |
| g-Gt | -269.333345 | 0.005261 | 3.30 |
| g-Gg | -269.332631 | 0.005975 | 3.75 |
| gTt | -269.332247 | 0.006359 | 3.99 |
| gTg- | -269.332297 | 0.006309 | 3.96 |
| gTg | -269.331907 | 0.006699 | 4.20 |
| gGt | -269.33162 | 0.006986 | 4.38 |

Table 4S. Total and relative zero-point corrected energies of 2ME conformers at BMK/6-31+G (d, p).

| Conformer | E_0_ (au) | Relative energy  (au) | Relative energy  (kcal/mol) |
| --- | --- | --- | --- |
| tGg- | -269.297948 | 0.0 | 0.0 |
| gGg- | -269.295366 | 0.002582 | 1.62 |
| tTt | -269.293994 | 0.003954 | 2.48 |
| tTg | -269.293638 | 0.00431 | 2.70 |
| tGt | -269.29269 | 0.005258 | 3.30 |
| tGg | -269.292203 | 0.005745 | 3.60 |
| g-Gt | -269.292243 | 0.005705 | 3.58 |
| g-Gg | -269.29178 | 0.006168 | 3.87 |
| gTt | -269.291283 | 0.006665 | 4.18 |
| gTg- | -269.291101 | 0.006847 | 4.30 |
| gTg | -269.290808 | 0.00714 | 4.48 |
| gGt | -269.290137 | 0.007811 | 4.90 |

.

Table 5S. Optimized transition states of 2ME pyrolysis at CBS-QB3 (left) and BMK/6-31+G (d, p) (right)

| 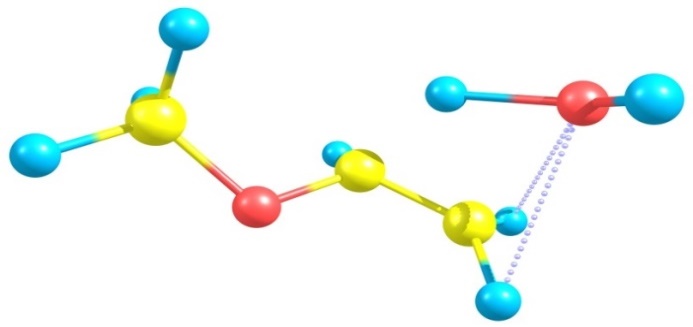  TS(methoxyethene+H2O) -2078.2572cm^-1^ at CBS-QB3  6 -1.219625000 -0.834315000 -0.160614000  1 -1.988088000 -1.344080000 0.403798000  1 -1.270547000 -0.931708000 -1.239427000  6 0.010662000 -0.495596000 0.454535000  1 -0.794421000 0.690614000 0.528640000  8 -1.954787000 0.881328000 0.064720000  1 -1.940432000 1.392564000 -0.759603000  1 0.246280000 -0.950724000 1.419194000  8 1.106940000 -0.386830000 -0.423340000  6 2.106419000 0.497062000 0.045425000  1 1.714984000 1.514563000 0.184588000  1 2.531960000 0.157281000 1.000573000  1 2.898309000 0.512595000 -0.704883000  CBS-QB3 (0 K)=-269.009547  CBS-QB3 Energy= -269.002818  CBS-QB3 Enthalpy= -269.001873  CBS-QB3 Free Energy= -269.039622 | 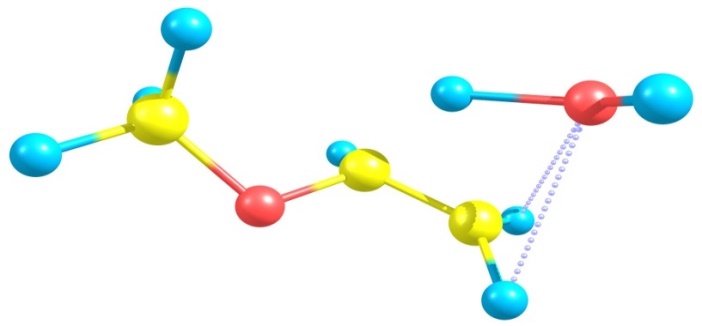  TS(methoxyethene+H2O) -1959.7079cm^-1^ at Bmk  6 -1.223537000 -0.823333000 -0.159109000  1 -1.984751000 -1.337778000 0.420617000  1 -1.294290000 -0.913665000 -1.242673000  6 0.023722000 -0.494628000 0.446376000  1 -0.822700000 0.698944000 0.527379000  8 -1.943817000 0.860773000 0.072862000  1 -2.005758000 1.409688000 -0.724370000  1 0.281732000 -0.976234000 1.396467000  8 1.091712000 -0.349638000 -0.443012000  6 2.111215000 0.476218000 0.053842000  1 1.741704000 1.497141000 0.247505000  1 2.530219000 0.074530000 0.992066000  1 2.902285000 0.508750000 -0.702446000  Sum of electronic and zero-point Energies= -269.187432  Sum of electronic and thermal Energies= -269.180867  Sum of electronic and thermal Enthalpies= -269.179923  Sum of electronic and thermal Free Energies -269.217546 |
| --- | --- |
| 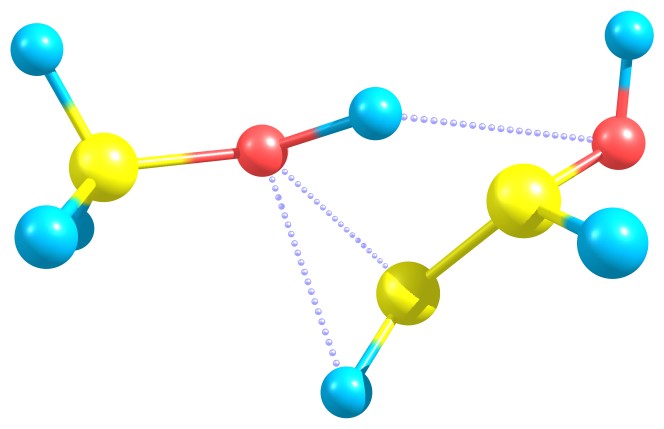  TS(methano+enol) -1968.6001cm^-1^  6 -1.154569000 0.102410000 0.510733000  1 0.024360000 -0.620818000 0.348647000  1 -1.348709000 0.328133000 1.561140000  6 -0.318085000 0.990838000 -0.199476000  1 -0.471130000 1.150104000 -1.258876000  1 0.275839000 1.726797000 0.330349000  8 1.057550000 -0.361974000 -0.370362000  6 2.359577000 -0.197057000 0.150492000  1 2.976001000 0.387624000 -0.541561000  1 2.360289000 0.309370000 1.130225000  1 2.837302000 -1.176098000 0.272323000  8 -2.268709000 -0.403748000 -0.196586000  1 -2.286215000 -1.356481000 -0.077156000  CBS-QB3 (0 K)= -269.008934  CBS-QB3 Energy= -269.001843  CBS-QB3 Enthalpy= -269.000899  CBS-QB3 Free Energy= -269.039416 | 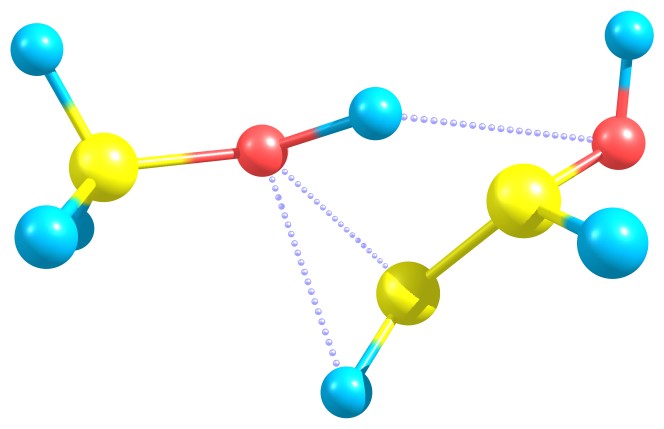  TS(methano+enol) -1890.3115cm^-1^  6 -1.135085000 0.110021000 0.520882000  1 0.103239000 -0.666371000 0.310230000  1 -1.336043000 0.414747000 1.552057000  6 -0.238131000 0.925030000 -0.233619000  1 -0.413571000 1.089896000 -1.295466000  1 0.374216000 1.667031000 0.280346000  8 1.031748000 -0.392331000 -0.400289000  6 2.299100000 -0.178915000 0.182801000  1 2.869585000 0.532521000 -0.427591000  1 2.207742000 0.219101000 1.208982000  1 2.850816000 -1.126794000 0.219161000  8 -2.268665000 -0.352498000 -0.171020000  1 -2.315950000 -1.308317000 -0.097633000  Sum of electronic and zero-point Energies= -269.187012  Sum of electronic and thermal Energies= -269.180236  Sum of electronic and thermal Enthalpies= -269.179292  Sum of electronic and thermal Free Energies-269.217024 |
| 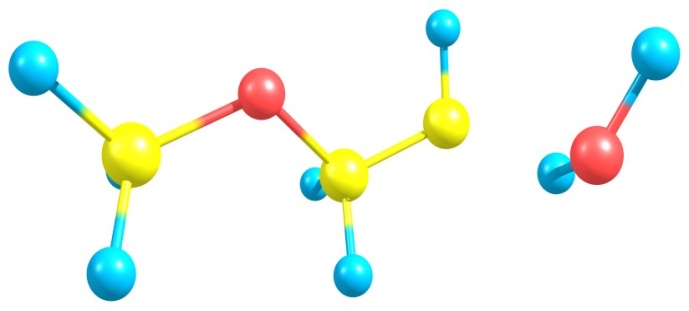  TS(methoxymethyl carbene +H2O) -823.9382cm^-1^  6 -1.180961000 0.877023000 -0.217580000  1 -2.318623000 0.308928000 0.370843000  1 -1.145488000 0.777608000 -1.307918000  6 0.143509000 0.609361000 0.354517000  1 0.566010000 1.628334000 0.492836000  1 0.061309000 0.172992000 1.360196000  8 0.995565000 -0.169555000 -0.472820000  6 2.278385000 -0.367717000 0.087952000  1 2.809923000 0.584177000 0.237565000  1 2.223617000 -0.890288000 1.053906000  1 2.845867000 -0.981405000 -0.612160000  8 -2.219308000 -0.755828000 0.184752000  1 -2.698271000 -0.909279000 -0.640059000  CBS-QB3 (0 K)= -268.993319  CBS-QB3 Energy= -268.986297  CBS-QB3 Enthalpy= -268.985353  CBS-QB3 Free Energy= -269.023869 | 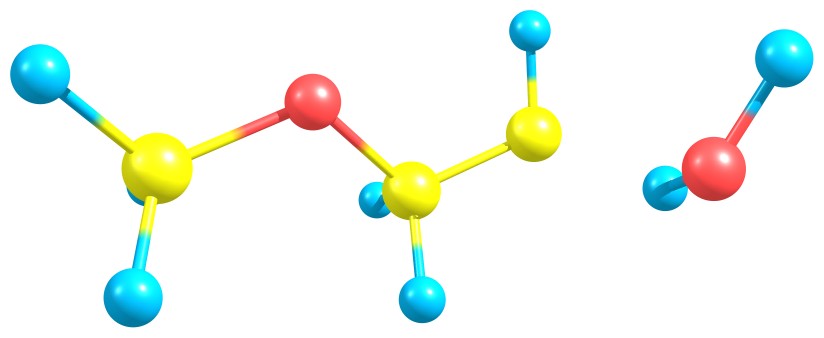  TS(methoxymethyl carbene +H2O) -769.2391cm^-1^  6 -1.225994000 0.856599000 -0.229253000  1 -2.387977000 0.230626000 0.401426000  1 -1.193700000 0.726881000 -1.321573000  6 0.133774000 0.647878000 0.332741000  1 0.558028000 1.675845000 0.374193000  1 0.081839000 0.281964000 1.372353000  8 0.953691000 -0.188312000 -0.449639000  6 2.240192000 -0.353786000 0.084593000  1 2.780928000 0.607286000 0.141004000  1 2.202861000 -0.794042000 1.095731000  1 2.785860000 -1.031333000 -0.579348000  8 -2.098718000 -0.756415000 0.198361000  1 -2.555450000 -1.043560000 -0.602055000  Sum of electronic and zero-point Energies= -269.169791  Sum of electronic and thermal Energies= -269.162915  Sum of electronic and thermal Enthalpies= -269.161971  Sum of electronic and thermal Free Energies -269.200127 |
| 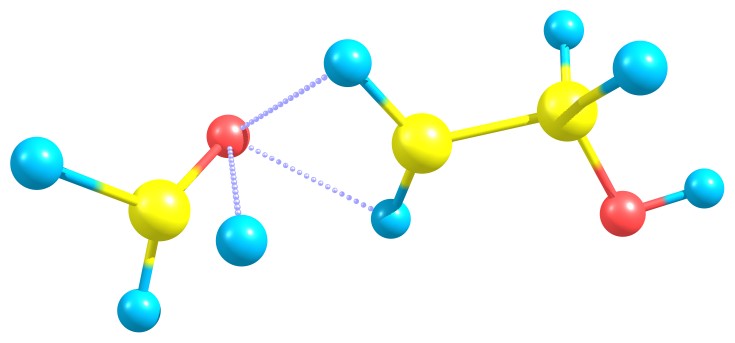  TS(ethanol+Fromaldhyde) -552.2613 cm^-1^  6 -1.662193000 -0.510220000 0.190045000  1 -1.812982000 -1.359175000 -0.506706000  1 -1.958685000 -0.887765000 1.184816000  6 -0.219030000 -0.226161000 0.196044000  1 1.357094000 0.484109000 1.045266000  1 0.454428000 -1.011396000 0.480893000  1 0.130545000 0.650567000 -0.315365000  8 -2.395044000 0.634545000 -0.194165000  1 -3.321861000 0.393731000 -0.278319000  8 1.986809000 -0.270068000 -0.739367000  6 2.338296000 0.343898000 0.369471000  1 2.994710000 -0.223862000 1.059346000  1 2.680195000 1.392870000 0.264959000  CBS-QB3 (0 K)= -268.987219  CBS-QB3 Energy= -268.980432  CBS-QB3 Enthalpy= -268.979488  CBS-QB3 Free Energy= -269.017456 | 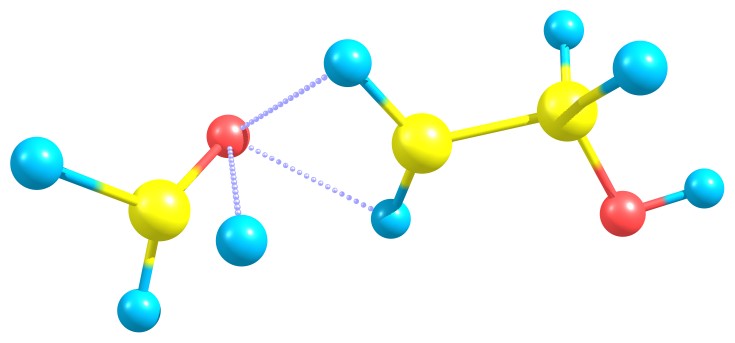  TS(ethanol+Fromaldhyde) -585.9087cm^-1^  6 1.651985000 0.524737000 0.151349000  1 1.807291000 1.321814000 -0.605535000  1 1.939643000 0.963671000 1.126317000  6 0.196103000 0.235717000 0.171483000  1 -1.340430000 -0.353560000 1.110677000  1 -0.476877000 1.057048000 0.359897000  1 -0.138462000 -0.714727000 -0.215549000  8 2.376742000 -0.638077000 -0.142880000  1 3.310785000 -0.432755000 -0.239649000  8 -1.953865000 0.177857000 -0.767785000  6 -2.321099000 -0.290744000 0.399321000  1 -2.981494000 0.367093000 1.001724000  1 -2.665407000 -1.345088000 0.414521000  Sum of electronic and zero-point Energies= -269.148774  Sum of electronic and thermal Energies= -269.142354  Sum of electronic and thermal Enthalpies= -269.141409 Sum of electronic and thermal Free Energies -269.178717 |
| 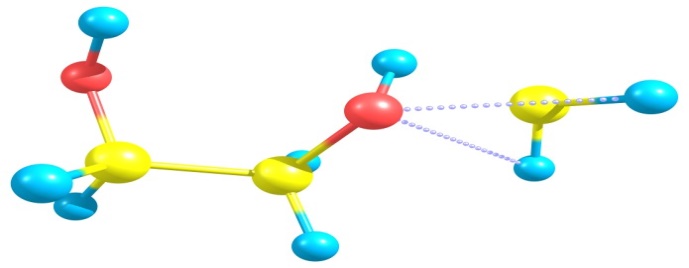  TS(EG+ methylene) -646.2520cm^-1^  6 1.453490000 0.497180000 0.187546000  1 2.081451000 1.251431000 -0.294223000  1 1.579073000 0.596010000 1.273582000  6 0.003417000 0.758834000 -0.183946000  1 -0.319523000 1.745741000 0.158658000  1 -0.137391000 0.685727000 -1.266344000  8 -0.835899000 -0.216092000 0.471667000  6 -2.533050000 -0.318672000 -0.270103000  1 -2.622986000 0.724055000 -0.608241000  1 -1.090651000 -0.948090000 -0.185380000  1 -3.072943000 -0.377271000 0.684196000  8 1.904263000 -0.766425000 -0.276134000  1 1.492918000 -1.441514000 0.272503000  CBS-QB3 (0 K)= -268.986347  CBS-QB3 Energy= -268.979313  CBS-QB3 Enthalpy= -268.978369  CBS-QB3 Free Energy= -269.016887 | 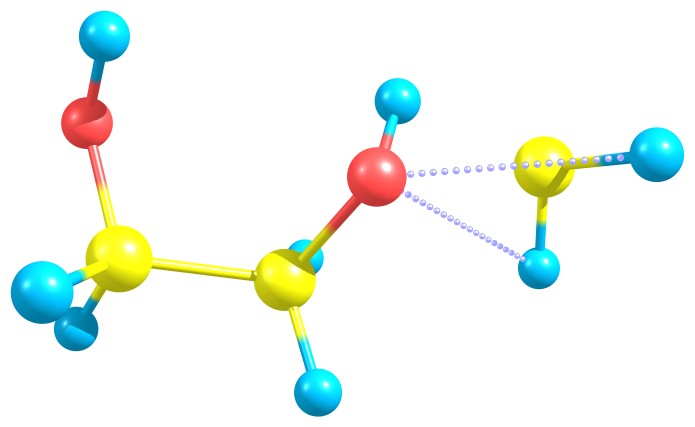  TS(EG+ methylene) -702.9670cm^-1^  6 1.426645000 0.503823000 0.218425000  1 2.045482000 1.295550000 -0.221375000  1 1.517656000 0.563234000 1.313888000  6 -0.029215000 0.740267000 -0.185855000  1 -0.392070000 1.703632000 0.195764000  1 -0.142360000 0.711474000 -1.278499000  8 -0.847986000 -0.276003000 0.397767000  6 -2.481147000 -0.287373000 -0.228710000  1 -2.558190000 0.763772000 -0.554449000  1 -1.079277000 -0.996572000 -0.276676000  1 -3.010676000 -0.370807000 0.732842000  8 1.916998000 -0.720298000 -0.272443000  1 1.569640000 -1.440176000 0.262757000  Sum of electronic and zero-point Energies= -269.162292  Sum of electronic and thermal Energies= -269.155543  Sum of electronic and thermal Enthalpies= -269.154598  Sum of electronic and thermal Free Energies -269.192311 |
| 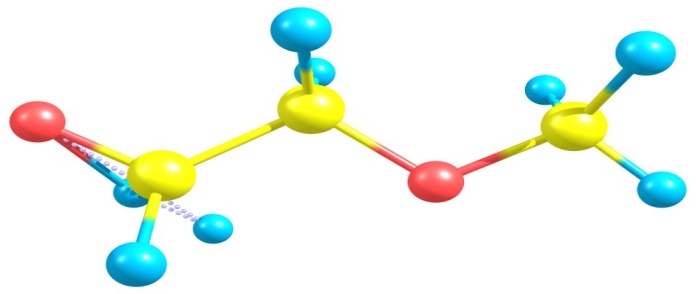  TS(methoxyethanal+H_2_) -2151.4539cm^-1^  6 -1.280701000 0.334171000 -0.165765000  1 -1.166023000 1.276962000 0.908312000  1 -1.211355000 1.099808000 -0.959053000  6 0.026194000 -0.414976000 0.011002000  1 0.010615000 -0.970132000 0.957848000  1 0.039850000 -1.151994000 -0.809847000  8 -2.418038000 -0.330683000 0.008293000  1 -1.972230000 0.702290000 0.846526000  8 1.103537000 0.486413000 -0.069392000  6 2.368793000 -0.140078000 0.043190000  1 2.475574000 -0.660230000 1.004899000  1 2.532506000 -0.862666000 -0.768093000  1 3.121359000 0.645418000 -0.022367000  CBS-QB3 (0 K)= -268.982473  CBS-QB3 Energy= -268.976068  CBS-QB3 Enthalpy= -268.975124  CBS-QB3 Free Energy= -269.012294 | 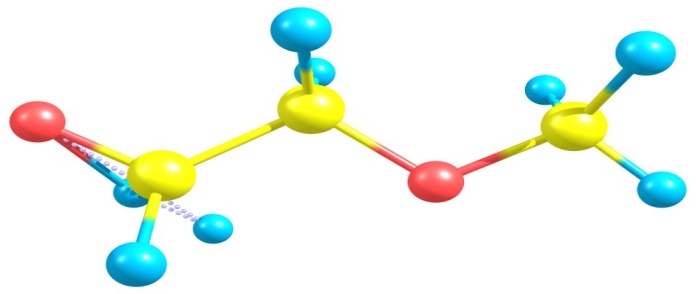  TS(methoxyethanal+H_2_) -2448.7177cm^-1^  6 -1.276652000 0.338514000 -0.169327000  1 -1.202733000 1.268118000 0.903492000  1 -1.212219000 1.102085000 -0.966742000  6 0.035856000 -0.411279000 0.028531000  1 0.015634000 -0.957655000 0.985445000  1 0.055776000 -1.156971000 -0.789623000  8 -2.410779000 -0.335607000 -0.003856000  1 -1.999194000 0.694084000 0.844313000  8 1.102117000 0.484710000 -0.051766000  6 2.359894000 -0.138151000 0.028156000  1 2.472484000 -0.688735000 0.976602000  1 2.509025000 -0.838927000 -0.809801000  1 3.115929000 0.650662000 -0.022874000  Sum of electronic and zero-point Energies= -269.159584  Sum of electronic and thermal Energies= -269.153250  Sum of electronic and thermal Enthalpies=-269.152306  Sum of electronic and thermal Free Energies -269.189404 |
| 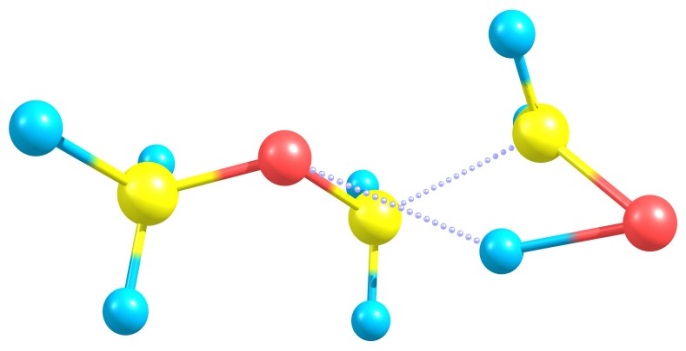  TS(dimethylether + fromaldhyde) -2097.7268cm^-1^  6 -1.588090000 0.139070000 -0.540509000  1 -1.851985000 1.143646000 -0.913530000  1 -1.123648000 -0.495376000 -1.311158000  6 0.158065000 0.591202000 0.259867000  1 0.285370000 1.577671000 -0.200745000  1 0.220909000 0.714153000 1.361853000  8 1.021876000 -0.385529000 -0.225052000  6 2.393219000 -0.129491000 0.048621000  1 2.720736000 0.815474000 -0.404764000  1 2.582946000 -0.089643000 1.129368000  1 2.961307000 -0.952329000 -0.384944000  8 -2.334121000 -0.405576000 0.409839000  1 -1.076839000 0.010559000 0.637743000  CBS-QB3 (0 K)= -268.975071  CBS-QB3 Energy= -268.968290  CBS-QB3 Enthalpy= -268.967346  CBS-QB3 Free Energy= -269.005858 | 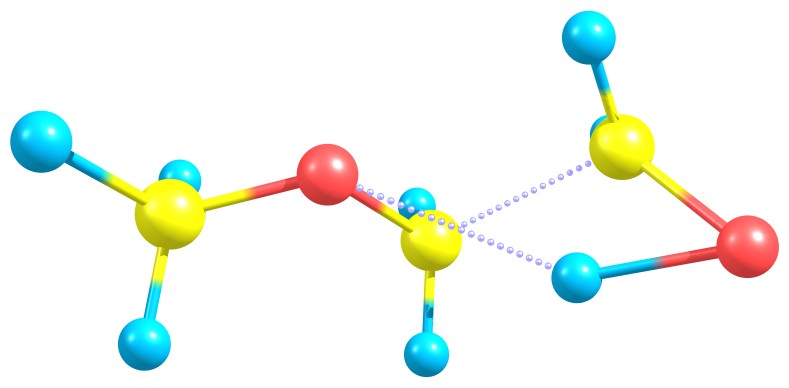  TS(dimethylether + fromaldhyde) -2237.4193cm^-1^  6 1.576262000 -0.442968000 0.350165000  1 1.834947000 -0.369921000 1.421088000  1 1.116982000 -1.405097000 0.067906000  6 -0.149265000 0.489014000 0.395621000  1 -0.277184000 0.558713000 1.486430000  1 -0.224013000 1.522792000 -0.019893000  8 -1.022337000 -0.389599000 -0.220105000  6 -2.375051000 -0.001540000 -0.137269000  1 -2.697673000 0.087250000 0.912730000  1 -2.541355000 0.961604000 -0.646411000  1 -2.963746000 -0.778919000 -0.632980000  8 2.320298000 0.210873000 -0.525585000  1 1.056675000 0.586349000 -0.274457000  Sum of electronic and zero-point Energies= -269.154751  Sum of electronic and thermal Energies= -269.148199  Sum of electronic and thermal Enthalpies= -269.147255  Sum of electronic and thermal Free Energies-269.185064 |
| 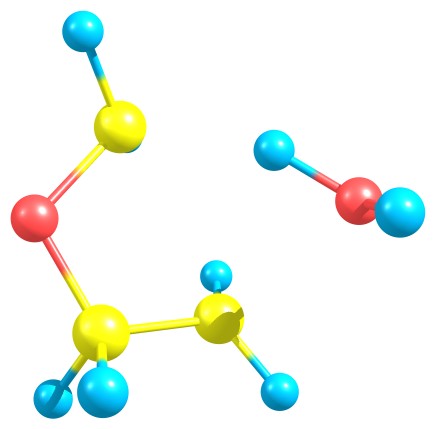  TS(Cycloxobutane+H2O) -545.8553cm^-1^  6 0.730294000 -0.998048000 -0.260265000  1 1.335991000 -1.822832000 0.169022000  1 0.509784000 -1.303690000 -1.293377000  6 -0.512687000 -0.955533000 0.546072000  1 -0.489681000 -0.538314000 1.540143000  1 -1.223278000 -1.760690000 0.401055000  8 -1.943910000 0.421347000 -0.217463000  1 -2.043124000 0.274712000 -1.170428000  8 1.445225000 0.188131000 -0.277852000  6 0.653177000 1.255010000 0.260097000  1 -1.062208000 0.957671000 -0.106998000  1 0.745208000 1.325481000 1.351959000  1 0.992090000 2.183263000 -0.204279000  CBS-QB3 (0 K)= -268.967407  CBS-QB3 Energy= -268.961049  CBS-QB3 Enthalpy= -268.960105  CBS-QB3 Free Energy= -268.996810 | 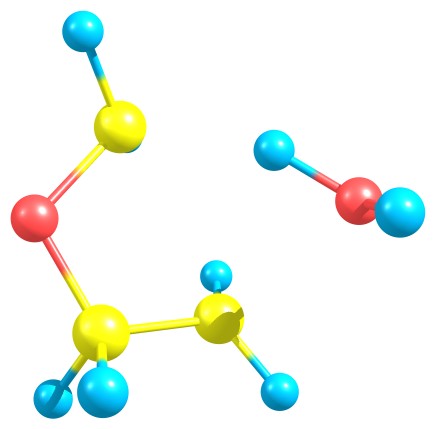  TS(Cycloxobutane+H2O) -751.0206cm^-1^  6 -0.864367000 -0.895908000 0.349787000  1 -1.451091000 -1.812936000 0.146999000  1 -0.729038000 -0.824738000 1.448040000  6 0.468604000 -0.962706000 -0.363351000  1 0.428728000 -0.696309000 -1.416472000  1 1.135875000 -1.792223000 -0.126055000  8 2.014343000 0.289515000 0.035027000  1 2.303319000 0.264293000 0.958296000  8 -1.500215000 0.214101000 -0.169620000  6 -0.540784000 1.244510000 -0.022323000  1 1.131079000 0.847341000 -0.026837000  1 -0.775692000 2.059093000 -0.716285000  1 -0.536918000 1.611179000 1.024374000  Sum of electronic and zero-point Energies= -269.138857  Sum of electronic and thermal Energies= -269.133102  Sum of electronic and thermal Enthalpies= -269.132158  Sum of electronic and thermal Free Energies-269.167473 |
| 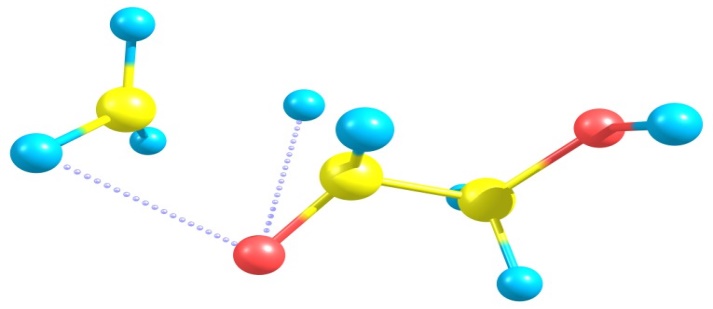  TS(Glycolaldhyde+methane) -1972.9792cm^-1^  6 -0.118341000 0.065237000 -0.189590000  1 -0.042802000 -0.486737000 -1.151870000  1 -0.647805000 -0.841382000 0.410351000  6 1.295062000 0.376494000 0.292146000  1 1.541664000 1.357667000 -0.146588000  1 1.304256000 0.498824000 1.376892000  8 2.258069000 -0.626459000 -0.007502000  1 2.381862000 -0.641905000 -0.961297000  8 -0.958195000 1.099864000 -0.142727000  6 -2.393951000 -0.692225000 0.113159000  1 -2.313439000 -1.769032000 0.008858000  1 -2.546297000 -0.270970000 1.095159000  1 -2.773050000 -0.130739000 -0.723965000  CBS-QB3 (0 K)= -268.964988  CBS-QB3 Energy= -268.957819  CBS-QB3 Enthalpy= -268.956875  CBS-QB3 Free Energy= -268.995576 | 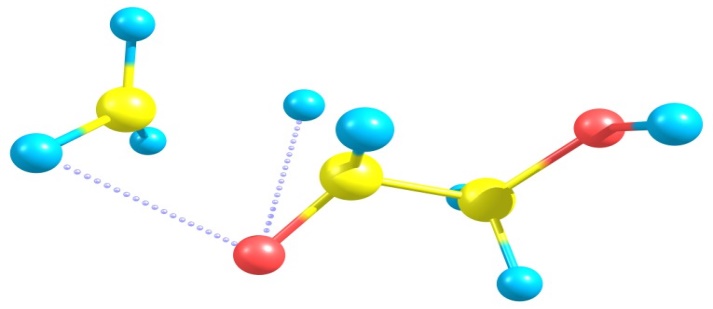  TS(Glycolaldhyde+methane) -1804.1580 cm^-1^  6 -0.182386000 0.049098000 -0.215681000  1 -0.089162000 -0.467164000 -1.199237000  1 -0.591577000 -0.847751000 0.460883000  6 1.231574000 0.375574000 0.309633000  1 1.423521000 1.385154000 -0.103019000  1 1.222770000 0.460724000 1.401104000  8 2.224245000 -0.563317000 -0.022500000  1 2.394004000 -0.530979000 -0.968461000  8 -1.041845000 1.084083000 -0.135067000  6 -2.166469000 -0.723734000 0.118300000  1 -2.046095000 -1.809043000 0.053622000  1 -2.434372000 -0.305348000 1.084106000  1 -2.634606000 -0.257354000 -0.741972000  Sum of electronic and zero-point Energies= -269.125899  Sum of electronic and thermal Energies= -269.119098  Sum of electronic and thermal Enthalpies= -269.118154  Sum of electronic and thermal Free Energies-269.156106 |
| 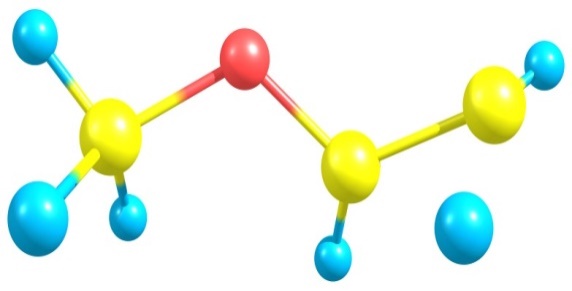Ts(methoxymethyl carbene to methoxyethene)  -1987.3432cm^-1^  6 -1.949963000 -0.112294000 0.146443000  1 -2.781399000 -0.515731000 -0.438949000  6 -0.614641000 0.355215000 -0.122371000  1 -0.477704000 1.231559000 -0.768722000  1 -1.326310000 0.709709000 0.961394000  8 0.443030000 -0.526524000 -0.080456000  6 1.713774000 0.098337000 0.070729000  1 1.899118000 0.819425000 -0.735858000  1 1.787660000 0.613869000 1.034827000  1 2.459377000 -0.694194000 0.022160000  CBS-QB3 (0 K)= -192.584736  CBS-QB3 Energy= -192.579369  CBS-QB3 Enthalpy= -192.578424  CBS-QB3 Free Energy= -192.613109 |  |

Table 6S. Optimized structures of products and radicals of 2ME pyrolysis at CBS-QB3.

| 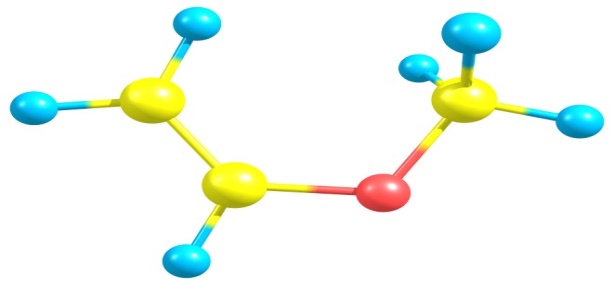  methoxyethene  6 0.000000000 0.932189000 0.000000000  1 0.491075000 1.899293000 0.000000000  6 -1.328646000 0.815258000 0.000000000  1 -1.848119000 -0.133052000 0.000000000  1 -1.931581000 1.712916000 0.000000000  8 0.932601000 -0.050756000 0.000000000  6 0.457687000 -1.390156000 0.000000000  1 -0.143791000 -1.595303000 0.892784000  1 -0.143791000 -1.595303000 -0.892784000  1 1.341156000 -2.026248000 0.000000000  CBS-QB3 (0 K)= -192.777633  CBS-QB3 Energy= -192.772856  CBS-QB3 Enthalpy= -192.771912  CBS-QB3 Free Energy= -192.804361 | 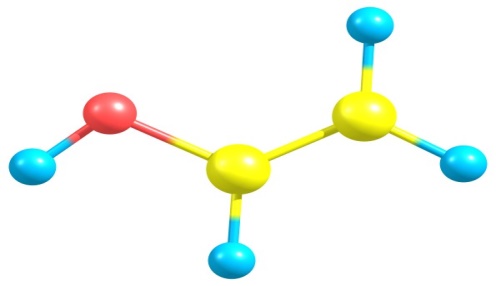  Vinyl alcohol  6 0.000000000 0.412845000 0.000000000  1 -0.194303000 1.483081000 0.000000000  6 1.236627000 -0.071499000 0.000000000  1 1.421111000 -1.138513000 0.000000000  1 2.081523000 0.602546000 0.000000000  8 -1.104163000 -0.393321000 0.000000000  1 -1.894786000 0.151378000 0.000000000  CBS-QB3 (0 K)= -153.564112  CBS-QB3 Energy= -153.560237  CBS-QB3 Enthalpy= -153.559293  CBS-QB3 Free Energy= -153.588770 |
| --- | --- |
| 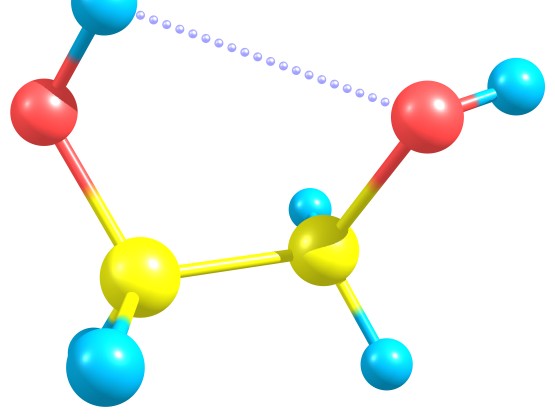  Ethylene glycol  6 -0.682712000 0.608540000 -0.266551000  1 -1.199114000 1.514504000 0.080518000  1 -0.655362000 0.611322000 -1.363529000  6 0.736347000 0.571722000 0.268754000  1 1.291753000 1.444781000 -0.082559000  1 0.704854000 0.605814000 1.367645000  8 1.427969000 -0.578291000 -0.189677000  1 0.843639000 -1.322080000 -0.000208000  8 -1.324700000 -0.574359000 0.222319000  1 -2.133741000 -0.714714000 -0.276227000  CBS-QB3 (0 K)= -229.911540  CBS-QB3 Energy= -229.906513  CBS-QB3 Enthalpy= -229.905569  CBS-QB3 Free Energy= -229.938764 | 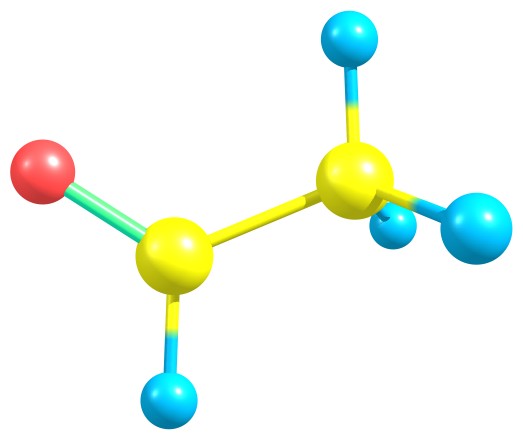  Acetaldhyde.  8 -1.201748000 0.390463000 0.000000000  6 0.000000000 0.461916000 0.000000000  6 0.930067000 -0.722745000 0.000000000  1 1.582600000 -0.679756000 0.878920000  1 0.362455000 -1.653003000 0.000000000  1 1.582600000 -0.679756000 -0.878920000  1 0.505930000 1.453785000 0.000000000  CBS-QB3 (0 K)= -153.582469  CBS-QB3 Energy= -153.578560  CBS-QB3 Enthalpy= -153.577616  CBS-QB3 Free Energy= -153.607419 |
| 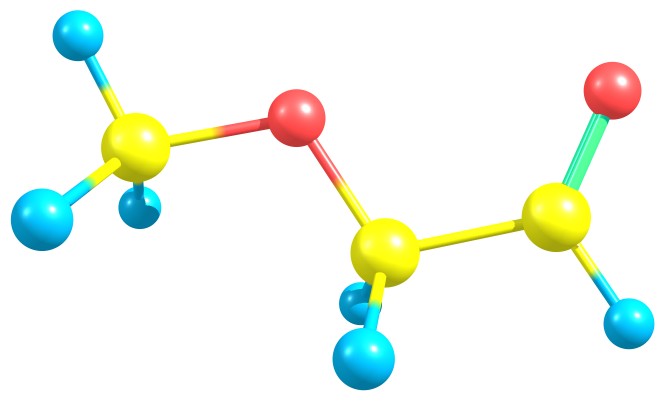  Methoxyethanal  8 -0.752589000 -0.460923000 0.000000000  6 0.000000000 0.714000000 0.000000000  1 -0.209260000 1.346822000 0.883191000  1 -0.209260000 1.346822000 -0.883191000  6 -2.147222000 -0.222264000 0.000000000  1 -2.462659000 0.336171000 0.892985000  1 -2.462659000 0.336171000 -0.892985000  1 -2.635622000 -1.196320000 0.000000000  6 1.484580000 0.423968000 0.000000000  8 1.984179000 -0.666123000 0.000000000  1 2.102596000 1.352483000 0.000000000  CBS-QB3 (0 K)= -267.926789  CBS-QB3 Energy= -267.920756  CBS-QB3 Enthalpy= -267.919811  CBS-QB3 Free Energy= -267.956171 | 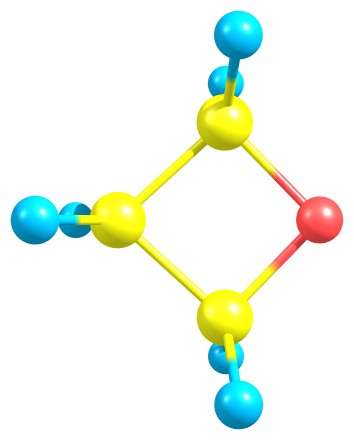  oxetane  6 -1.038601000 -0.063721000 0.000009000  6 -0.000186000 1.076157000 -0.000011000  8 0.000141000 -1.072280000 -0.000004000  1 -1.669617000 -0.132079000 0.892187000  1 -1.669577000 -0.132090000 -0.892196000  1 -0.000196000 1.705881000 0.889694000  1 -0.000268000 1.705912000 -0.889694000  6 1.038674000 -0.063472000 -0.000003000  1 1.669559000 -0.131544000 0.892273000  1 1.669649000 -0.131621000 -0.892205000  CBS-QB3 (0 K)= -192.766697  CBS-QB3 Energy= -192.762589  CBS-QB3 Enthalpy= -192.761645  CBS-QB3 Free Energy= -192.793223 |
| 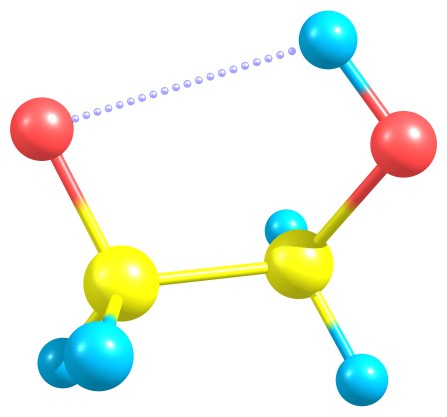  EG-r1  6 -0.679083000 0.608530000 -0.232027000  1 -1.161714000 1.474799000 0.226821000  1 -0.703538000 0.743842000 -1.323396000  6 0.787605000 0.518224000 0.219277000  1 1.387463000 1.402303000 -0.055600000  1 0.798514000 0.467051000 1.328928000  8 1.376770000 -0.652286000 -0.168022000  8 -1.392393000 -0.541877000 0.167073000  1 -0.846869000 -1.295222000 -0.092660000  CBS-QB3 (0 K)= -229.244383  CBS-QB3 Energy= -229.239738  CBS-QB3 Enthalpy= -229.238794  CBS-QB3 Free Energy= -229.271932 | 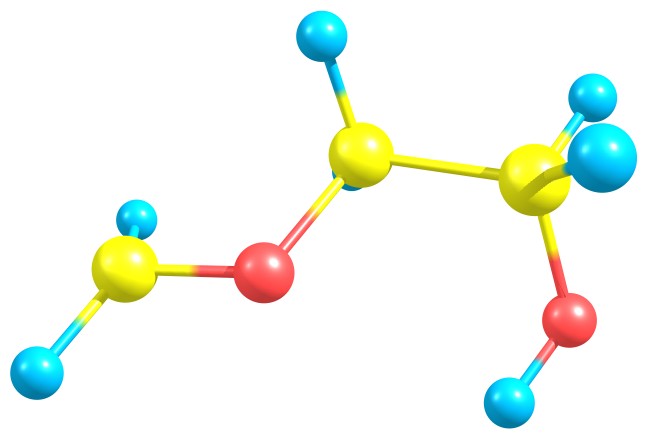  2ME- r4  6 -1.338546000 0.467468000 -0.251806000  1 -2.017919000 1.234549000 0.127192000  1 -1.379730000 0.487881000 -1.349862000  6 0.075288000 0.778380000 0.197807000  1 0.406406000 1.756355000 -0.172570000  1 0.138065000 0.764894000 1.292891000  8 0.917625000 -0.251410000 -0.334705000  6 2.217032000 -0.216339000 0.059014000  1 2.455513000 0.261103000 1.004768000  1 2.806482000 -1.037579000 -0.323597000  8 -1.791468000 -0.775035000 0.259888000  1 -1.140713000 -1.432695000 -0.010373000  CBS-QB3 (0 K)= -268.471444  CBS-QB3 Energy= -268.465004  CBS-QB3 Enthalpy= -268.464059  CBS-QB3 Free Energy= -268.501711 |
| 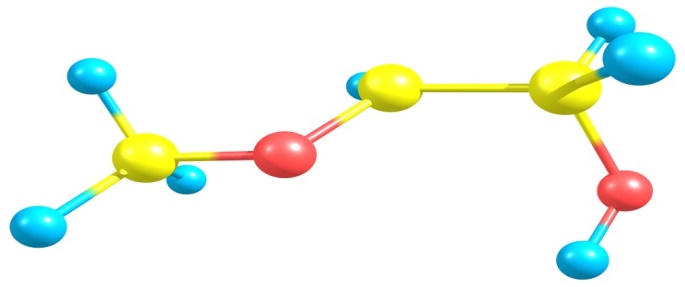  2ME-r3  6 -1.355761000 0.533570000 -0.229566000  1 -1.944514000 1.364047000 0.162608000  1 -1.373100000 0.585206000 -1.326303000  6 0.044499000 0.640762000 0.246544000  1 0.270027000 0.852727000 1.290475000  8 0.921987000 -0.171226000 -0.411887000  6 2.246289000 -0.193511000 0.108666000  1 2.703498000 0.799636000 0.047408000  1 2.250560000 -0.530566000 1.151930000  1 2.814218000 -0.894488000 -0.501370000  8 -2.026344000 -0.661087000 0.225093000  1 -1.495990000 -1.402985000 -0.084260000  CBS-QB3 (0 K)= -268.472933  CBS-QB3 Energy= -268.466214  CBS-QB3 Enthalpy= -268.465270  CBS-QB3 Free Energy= -268.503804 | 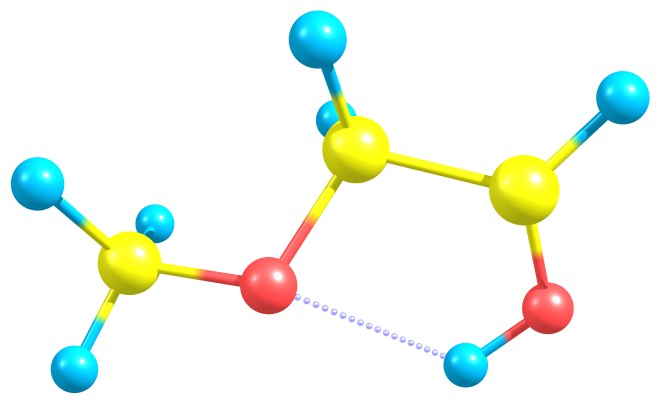  2ME-r2  6 -1.432074000 0.495704000 -0.183358000  1 -2.203146000 1.252257000 -0.114742000  6 -0.010535000 0.814900000 0.119517000  1 0.295206000 1.741919000 -0.386204000  1 0.160057000 0.956886000 1.206950000  8 0.770970000 -0.299402000 -0.313112000  6 2.126576000 -0.225776000 0.085340000  1 2.627171000 0.646340000 -0.356854000  1 2.223852000 -0.168967000 1.178669000  1 2.616537000 -1.132163000 -0.270913000  8 -1.863253000 -0.759660000 0.131428000  1 -1.085218000 -1.332748000 0.067575000  CBS-QB3 (0 K)= -268.473471  CBS-QB3 Energy= -268.467095  CBS-QB3 Enthalpy= -268.466150  CBS-QB3 Free Energy=-268.503698 |
| 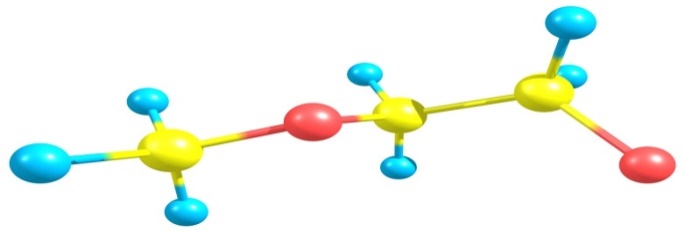  2ME- r1  6 -1.408437000 0.387899000 -0.252264000  1 -2.069328000 1.238957000 0.008629000  1 -1.409540000 0.369524000 -1.357461000  6 0.000698000 0.633333000 0.267839000  1 0.305279000 1.673246000 0.060763000  1 0.013608000 0.481574000 1.357644000  8 0.865251000 -0.276912000 -0.379757000  6 2.198314000 -0.201601000 0.083340000  1 2.630492000 0.795611000 -0.086384000  1 2.266485000 -0.433932000 1.155532000  1 2.775741000 -0.937435000 -0.476663000  8 -2.022275000 -0.736254000 0.222813000  CBS-QB3 (0 K)= -268.454431  CBS-QB3 Energy= -268.448041  CBS-QB3 Enthalpy= -268.447097  CBS-QB3 Free Energy= -268.484876 | 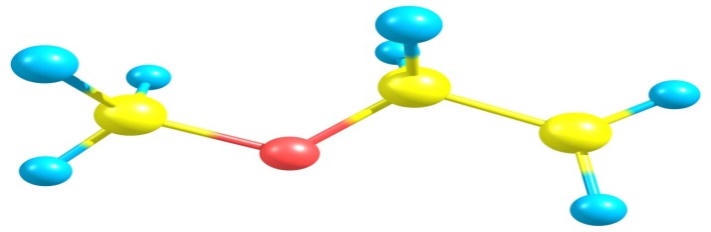  2Ethoxymethane-r1  6 1.892908000 -0.181095000 -0.009210000  1 2.790268000 0.406562000 -0.151079000  1 1.972280000 -1.231086000 0.237333000  6 0.566265000 0.475535000 0.020953000  1 0.441859000 1.065909000 0.953235000  1 0.471462000 1.203308000 -0.806317000  8 -0.452582000 -0.504579000 -0.060079000  6 -1.749772000 0.046830000 0.013487000  1 -1.911265000 0.582182000 0.961148000  1 -1.943960000 0.743289000 -0.815788000  1 -2.456396000 -0.781150000 -0.049279000  CBS-QB3 (0 K)= -193.321440  CBS-QB3 Energy= -193.315435  CBS-QB3 Enthalpy= -193.314491  CBS-QB3 Free Energy= -193.350349 |
| 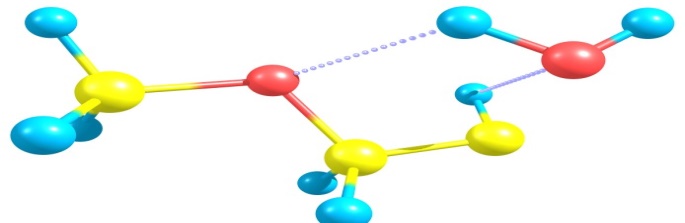  Methoxymethyl carbene-----H2O adduct  6 -1.419269000 0.875445000 -0.134575000  1 -2.435865000 -1.140385000 -0.291181000  1 -1.429493000 0.721860000 -1.228383000  6 0.008404000 0.881144000 0.259250000  1 0.409114000 1.859029000 -0.049432000  1 0.151210000 0.776255000 1.343432000  8 0.761217000 -0.198179000 -0.390298000  6 2.098524000 -0.324983000 0.060093000  1 2.686725000 0.568795000 -0.187451000  1 2.149102000 -0.484548000 1.146485000  1 2.539488000 -1.185672000 -0.445811000  8 -1.678177000 -0.859543000 0.233877000  1 -0.860564000 -1.243195000 -0.144897000  CBS-QB3 (0 K) -268.997550  CBS-QB3 Energy= -268.990521  CBS-QB3 Enthalpy= -268.989576  CBS-QB3 Free Energy= -269.027588 | 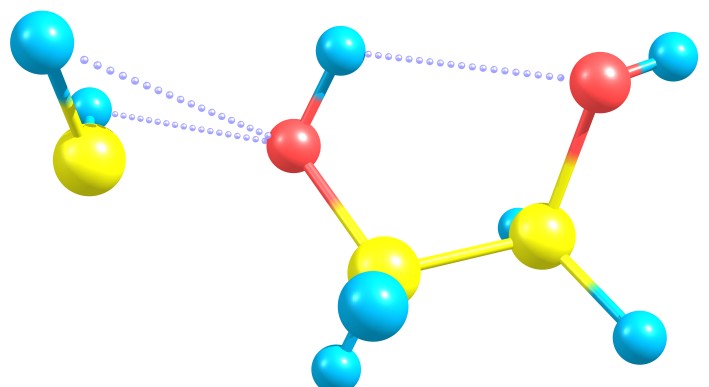  EG---CH2 adduct  6 -1.408980000 0.527629000 -0.186120000  1 -2.073198000 1.251572000 0.301862000  1 -1.503247000 0.633745000 -1.273323000  6 0.019276000 0.788644000 0.236981000  1 0.402594000 1.745704000 -0.108034000  1 0.134798000 0.727745000 1.321247000  8 0.856219000 -0.216279000 -0.376167000  1 0.438018000 -1.072838000 -0.194207000  8 -1.695950000 -0.813732000 0.224985000  1 -2.514546000 -1.103926000 -0.186777000  6 2.468364000 -0.069837000 0.153755000  1 2.914016000 -0.510293000 -0.755458000  1 2.447453000 -0.910235000 0.876458000  CBS-QB3 (0 K)= -268.991034  CBS-QB3 Energy= -268.983894  CBS-QB3 Enthalpy= -268.982950  CBS-QB3 Free Energy= -269.021487 |

Table 7S. Isodesmic equations used in calculation of enthalpy of formation.

| CH_3_OCH_2_CH_2_OH + CH_3_CH_2_CH_3_  → CH_3_OCH_2_CH_3_ + CH_3_CH_2_CH_2_OH  CH_3_OCH_2_CH_2_OH + CH_3_CH_3_ →CH_3_OCH_2_CH_3_ + CH_3_CH_2_OH  CH_3_OCH_2_CH_2_OH + CH_3_OCH_3_ →CH3OCH_2_CH_2_OCH_3_ + CH_3_OH  CH_3_OCH_2_CH_2_OH + CH_3_CH_2_OH → CH3OCH_2_CH_3_ + HOCH_2_CH_2_OH  CH_3_OCH_2_CH_2_OH + CH_3_CH_2_CH_2_OH → CH_3_OCH_2_CH_3_ + HOCH_2_CH_2_CH_2_OH  CH_3_OCH_2_CH_2_OH +CH_3_OH → CH_3_OCH_3_+ HOCH_2_CH_2_OH  CH_3_OCH_2_CH_2_OH + CH_3_CH_2_CH_3_ → CH_3_OCH_3_+ CH_3_CH_2_CH_2_CH_2_OH |
| --- |
| CH_3_OCH_2_OCH_3_ + CH3CH2OH→ CH_3_OCH_2_CH_2_OH + CH_3_OCH_3_  CH_3_OCH_2_OCH_3_ + CH_3_CH_2_CH_2_OH → CH_3_OCH_2_CH_2_OH + CH_3_OCH_2_CH_3_  CH_3_OCH_2_CH_2_OH + CH_3_OCH_2_CH_2_OH → CH_3_OCH_2_OCH_3_ + HOCH_2_CH_2_CH_2_OH |
| HOCH_2_CH_2_OH + CH_3_CH_3_ → 2 CH_3_CH_2_OH  HOCH_2_CH_2_OH + CH_3_CH_2_CH_3_ → CH_3_CH_2_CH_2_OH+ CH_3_CH_2_OH  HOCH_2_CH_2_OH + CH_2_=CH-CH_3_ → CH_2_=CH-CH_2_OH + CH_3_CH_2_OH |
| CH_3_OCH_2_CHO + CH_3_CH_3_ → CH_3_OCH_3_+ CH_3_CH_2_CHO  CH_3_OCH_2_CHO +CH_4_ →CH_3_OH+ CH_3_CH_2_CHO  CH_3_OCH_2_CHO +CH_4_ → CH_3_CHO+ CH_3_OCH_3_  CH_3_OCH_2_CHO +CH_3_OCH_2_CH_3_ → CH_3_OCH_2_CH_2_OCH_3_+ CH_3_CHO |
| HOCH_2_CHO + CH_3_CH_3_ → CH_3_CH_2_OH+ CH_3_CHO  HOCH_2_CHO + CH_3_OCH_3_  →CH_3_OCHO + CH_3_CH_2_OH  HOCH_2_CHO + CH_3_OCH_2_CH_3_ → CH_3_OCHO + CH_3_CH_2_CH_2_OH  HOCH_2_CHO + CH_3_CH_2_CH_3_ → CH_3_CHO + CH_3_CH_2_CH_2_OH  HOCH_2_CHO + CH3OH → CH_3_OOH + CH_3_CHO |
| CH_3_OCH=CH_2_+ CH_3_CH_3_ →CH_3_OCH_3_+ CH_3_-CH=CH_2_  CH_3_OCH=CH_2_+CH_3_CH_2_OH→CH_3_OCH_2_CH_3_+ CH_2_=CH-OH  CH_3_OCH=CH_2_+CH_3_CH_2_OH→ CH_3_OCH_3_+ CH_2_=CH-CH_2_-OH  CH_3_OCH=CH_2_+CH_3_CH_2_CH_3_→CH_3_OCH_2_CH_3_+ CH_2_=CH-CH_3_  CH_3_OCH=CH_2_+CH_3_CH_2_CHO→CH_3_OCHO+ CH_2_=CH-CH_2_-CH_3_ |
| CH_2_=CHOH +CH_3_CH_3_→ CH_3_-CH=CH_2_+ CH_3_OH  CH_2_=CHOH +CH_3_CH_2_CH_2_OH→ CH_3_-CH=CH_2_+ HOCH_2_CH_2_OH  CH_2_=CHOH + CH_3_CH_2_CH_3_→ CH_3_CH_2_OH+ CH_3_-CH=CH_2_  CH_2_=CHOH + CH_3_OH → CH_3_OOH+ CH_2_=CH_2_  CH_2_=CHOH + HCHO →HCOOH+ CH_2_=CH_2_ |
| CH_3_OCH_3_+ CH_4_→ C_2_H_6_+ CH_3_OH  CH_3_OCH_3_+ CH_3_OH → C_2_H_6_+ CH_3_OOH  CH_3_OCH_3_+CH_3_CH_2_CH_3_→ C_2_H_6_ +CH_3_OCH_2_CH_3_  CH_3_OCH_3_+HCOH→CH_4_+CH_3_OCOH  CH_3_OCH_3_+HCOH→ CH_4_+CH_3_OCOH |
| CH_3_OH+ CH_3_CH_2_CH_3_ → C_2_H_5_OH+ C_2_H_6_  CH_3_OH+CH_3_CH_2_CH_2_OH → 2 C_2_H_5_OH  CH_3_OH+CH_3_CH_2_CH_2_CH_2_OH → C_2_H_5_OH + C_3_H_7_OH  CH_3_OH+CH_3_CH_2_OCH_3_ → C_2_H_5_OH + CH_3_OCH_3_ |
| HCHO+ CH_3_CH_2_CH_3_ → CH_3_CH_2_CHO+ CH_4_  HCHO+ CH_3_CH_2_CH_2_OH → CH_3_CH_2_CHO + CH_3_OH  HCHO+ CH_3_CH_2_OCH_3_ → CH_3_CH_2_CHO + CH_3_OH  HCHO+CH_3_CH_3_ → CH_3_CHO+ CH_4_  HCHO+CH_3_OCH_3_  → CH_3_OCHO+ CH_4_ |
| CH_4_+CH_3_OCH_2_CH_3_ → CH_3_OCH_3_+CH_3_CH_2_CH_3_  CH_4_+CH_3_CH_2_CH_3_ → 2 C_2_H_6_  CH_4_+CH_3_CH_2_CH_2_OH → C_2_H_5_OH+C_2_H_6_  CH_4_+C_2_H_5_OOH → CH_3_OOH+C_2_H_6_ |
| H_2_O +CH_3_CH_3_ → CH_3_OH +CH_4_  H_2_O +CH_3_OCH_3_→ CH_3_OOH+ CH_4_  H_2_O + CH_3_OCH_3_→2 CH_3_OH  H_2_O+CH_3_CH_2_OCH_3_→CH_3_OH+C_2_H_5_OH |
| CH_3_OCH_2_CH_2_O•+CH_3_OH → CH_3_O• + CH_3_OCH_2_CH_2_OH  CH_3_OCH_2_CH_2_O•+CH_3_CH_2_OH →CH_3_CH_2_O•+ CH_3_OCH_2_CH_2_OH  CH_3_OCH_2_CH_2_O•+C_2_H_5_OOH → C_2_H_5_OO• + CH_3_OCH_2_CH_2_OH  CH_3_OCH_2_CH_2_O•+CH_3_CH_2_CH_2_OH →CH_3_CH_2_CH_2_O•+ CH_3_OCH_2_CH_2_OH  CH_3_OCH_2_CH_2_O•+(CH_3_)_2_CHOH →( CH_3_)_2_CHO•+CH_3_OCH_2_CH_2_OH |
| CH_3_OCH_2_CHOH +CH_3_CH_2_OH → CH_3_OCH_2_CH_2_OH +CH3CHOH  CH_3_OCH_2_CHOH + CH_3_CH_2_COOCH_2_CH_3_→ CH_3_OCH_2_CH_2_OH + CH_3_CHCOOCH_2_CH_3_  CH_3_OCH_2_CHOH +CH_3_OH → CH_3_OCH_2_CH_2_OH +•CHOH  CH_3_OCH_2_CHOH +CH_3_CH_2_CH_3_→ CH_3_OCH_2_CH_2_OH + CH_3_CHCH_3_ |
| CH_3_OCHCH_2_OH+CH_3_CH_2_CH_3_ → CH_3_OCH_2_CH_2_OH + CH_3_CHCH_3_  CH_3_OCHCH_2_OH +CH_3_CH_2_COOCH_2_CH_3_→ CH_3_OCH_2_CH_2_OH + CH_3_CHCOOCH_2_CH_3_  CH_3_OCHCH_2_OH +CH_3_CH_2_OH→CH_3_OCH_2_CH_2_OH +CH_3_CHOH |
| •CH_2_OCH_2_CH_2_OH + CH_3_OH → CH_3_OCH_2_CH_2_OH + •CH_2_OH  •CH_2_OCH_2_CH_2_OH + CH_3_CH_2_OH →CH_3_OCH_2_CH_2_OH + •CH_2_CH_2_OH  •CH_2_OCH_2_CH_2_OH +CH_3_OCH_3_ →CH_3_OCH_2_CH_2_OH + •CH_2_OCH_3_  •CH_2_OCH2CH2OH+CH_3_CHO → CH_3_OCH_2_CH_2_OH + •CH_2_CHO |
| •OCH_2_CH_2_OH+CH_3_CH_2_OH →HOCH_2_CH_2_OH+ CH_3_CH_2_O•  •OCH_2_CH_2_OH +CH_3_OH →HOCH_2_CH_2_OH + CH_3_O•  •OCH_2_CH_2_OH +CH_3_CH_2_OOH→ HOCH_2_CH_2_OH + CH_3_CH_2_OO•  •OCH_2_CH_2_OH +(CH_3_)_2_CHOH→HOCH_2_CH_2_OH +(CH3)_2_CHO• |
| CH_3_OCH_2_•CH_2_  CH_3_OCH_2_•CH_2_+CH_3_CH_3_→ CH_3_OCH_2_CH_3_+ •CH_2_CH_3_  CH_3_OCH_2_•CH_2_+ CH_3_CH_2_CH_3_→ •CH_2_CH_2_CH_3_+ CH_3_CH_2_OCH  CH_3_OCH_2_•CH_2_+CH_3_CH_2_OH→ •CH_2_CH_2_OH+ CH_3_CH_2_OCH_3_  CH_3_OCH_2_•CH_2_+ (CH3)_2_CHCH_3_→ (CH3)_2_CH•CH_2_+ CH_3_CH_2_OCH_3_ |

Table 8S. Barrier heights and relative energies of different channels of 2ME pyrolysis at BMK/6-31+G(d,p).

| Species | Energy | Species | Energy |
| --- | --- | --- | --- |
| TS1 | 73.47 | CH_3_+OCH_2_CH_2_OH | 81.12 |
| Methoxyethene +H_2_O | 9.45 | CH_3_OCH_2_+CH_2_OH | 83.05 |
| TS2 | 73.87 | CH_3_O+CH_2_CH_2_OH | 85.00 |
| Vinyl alcohol +Methanol | 15.4 | CH_3_OCH_2_CHOH+H | 93.67 |
| TS3 | 84.73 | CH_3_OCHCH_2_OH+H | 94.36 |
| TS4 | 97.64 | CH_2_OCH_2_CH_2_OH+H | 95.29 |
| Ethanol+ Formaldehyde | 6.91 | CH_3_OCH_2_CH_2_+OH | 95.72 |
| TS5 | 89.36 | CH_3_OCH_2_CH_2_O+H | 102.86 |
| EG+CH_2_ | 90.08 |  |  |
| TS6 | 90.80 |  |  |
| 2-Methoxyacetaldhyde+H_2_ | 22.36 |  |  |
| TS7 | 93.97 |  |  |
| DME+ Formaldehyde | 17.49 |  |  |
| TS8 | 103.44 |  |  |
| Oxetane + H_2_O | 12.15 |  |  |
| TS9 | 112.23 |  |  |
| Glycolaldhyde+CH_4_ | -2.45 |  |  |

Table 9S. Arrhenius coefficient for C- H and C_α_-O_α_ bonds fission reactions.

| Reaction | E_a_(kcal/mol) | A(s^-1^) |
| --- | --- | --- |
| C1-O1 | 98.42 | 4.58x10^22^ |
| C1-H1 | 97.31 | 1.19x10^21^ |
| C2-H3 | 97.96 | 3.92x10^21^ |
| C3-H7 | 98.74 | 1.59x10^21^ |

Table 10S. Rate constants (k, s^-1^) for 17 unimolecular decomposition reactions of 2-methoxyethanol at CBS-QB3.

| T | R1 | R2 | R3 | R4 | R5 |
| --- | --- | --- | --- | --- | --- |
| 298 | 3.17E-33 | 2.02E-34 | 3.67E-47 | 4.78E-50 | 2.77E-50 |
| 300 | 5.21E-33 | 3.51E-34 | 9.29E-47 | 1.27E-49 | 7.36E-50 |
| 400 | 3.03E-24 | 1.06E-24 | 1.08E-31 | 7.62E-34 | 5.86E-34 |
| 500 | 1.50E-17 | 1.31E-17 | 1.37E-22 | 2.51E-24 | 2.28E-24 |
| 600 | 1.44E-12 | 1.66E-12 | 1.72E-16 | 5.95E-18 | 6.08E-18 |
| 700 | 6.84E-09 | 9.08E-09 | 4.04E-12 | 2.21E-13 | 2.46E-13 |
| 800 | 4.34E-06 | 6.27E-06 | 7.99E-09 | 6.03E-10 | 7.12E-10 |
| 900 | 6.94E-04 | 1.07E-03 | 2.96E-06 | 2.90E-07 | 3.58E-07 |
| 1000 | 4.13E-02 | 6.67E-02 | 3.42E-04 | 4.13E-05 | 5.22E-05 |
| 1100 | 1.20E+00 | 2.00E+00 | 1.68E-02 | 2.38E-03 | 3.09E-03 |
| 1200 | 2.00E+01 | 3.45E+01 | 4.31E-01 | 7.11E-02 | 9.37E-02 |
| 1300 | 2.19E+02 | 3.86E+02 | 6.82E+00 | 1.26E+00 | 1.69E+00 |
| 1400 | 1.72E+03 | 3.07E+03 | 7.23E+01 | 1.49E+01 | 2.02E+01 |
| 1500 | 1.03E+04 | 1.88E+04 | 5.66E+02 | 1.26E+02 | 1.73E+02 |
| 1600 | 4.94E+04 | 9.19E+04 | 3.43E+03 | 8.18E+02 | 1.13E+03 |
| 1700 | 1.98E+05 | 3.73E+05 | 1.67E+04 | 4.30E+03 | 5.96E+03 |
| 1800 | 6.86E+05 | 1.29E+06 | 6.88E+04 | 1.88E+04 | 2.59E+04 |
| 1900 | 2.07E+06 | 3.96E+06 | 2.45E+05 | 7.05E+04 | 9.74E+04 |
| 2000 | 5.62E+06 | 1.09E+07 | 7.67E+05 | 2.32E+05 | 3.21E+05 |

Table 10S. Continued

| T | R6 | R7 | R8 | R9 | R10 | R11 |
| --- | --- | --- | --- | --- | --- | --- |
| 298 | 6.92E-45 | 1.95E-48 | 1.50E-59 | 1.25E-53 | 1.31E-41 | 4.56E-43 |
| 300 | 1.33E-44 | 4.35E-48 | 4.56E-59 | 2.87E-53 | 3.45E-41 | 1.25E-42 |
| 400 | 3.04E-33 | 8.91E-36 | 4.53E-41 | 2.02E-39 | 1.75E-25 | 3.18E-26 |
| 500 | 4.46E-25 | 5.93E-27 | 3.21E-30 | 1.52E-29 | 4.98E-16 | 2.34E-16 |
| 600 | 5.52E-19 | 1.86E-20 | 5.88E-23 | 1.91E-22 | 1.03E-09 | 9.09E-10 |
| 700 | 1.71E-14 | 1.10E-15 | 9.52E-18 | 2.88E-17 | 3.36E-05 | 4.65E-05 |
| 800 | 4.45E-11 | 4.56E-12 | 7.93E-14 | 2.42E-13 | 8.16E-02 | 1.58E-01 |
| 900 | 2.13E-08 | 3.12E-09 | 9.03E-11 | 2.87E-10 | 3.48E+01 | 8.73E+01 |
| 1000 | 3.05E-06 | 5.97E-07 | 2.57E-08 | 8.52E-08 | 4.41E+03 | 1.36E+04 |
| 1100 | 1.81E-04 | 4.47E-05 | 2.62E-06 | 9.14E-06 | 2.29E+05 | 8.34E+05 |
| 1200 | 5.53E-03 | 1.66E-03 | 1.25E-04 | 4.53E-04 | 6.12E+06 | 2.57E+07 |
| 1300 | 1.01E-01 | 3.53E-02 | 3.29E-03 | 1.26E-02 | 9.75E+07 | 4.62E+08 |
| 1400 | 1.22E+00 | 4.91E-01 | 5.53E-02 | 2.16E-01 | 1.04E+09 | 5.46E+09 |
| 1500 | 1.06E+01 | 4.85E+00 | 6.35E-01 | 2.59E+00 | 8.07E+09 | 4.62E+10 |
| 1600 | 7.17E+01 | 3.62E+01 | 5.34E+00 | 2.25E+01 | 4.80E+10 | 2.97E+11 |
| 1700 | 3.83E+02 | 2.13E+02 | 3.53E+01 | 1.54E+02 | 2.30E+11 | 1.53E+12 |
| 1800 | 1.72E+03 | 1.03E+03 | 1.89E+02 | 8.44E+02 | 9.21E+11 | 6.51E+12 |
| 1900 | 6.58E+03 | 4.25E+03 | 8.44E+02 | 3.90E+03 | 3.18E+12 | 2.37E+13 |
| 2000 | 2.21E+04 | 1.51E+04 | 3.31E+03 | 1.55E+04 | 9.63E+12 | 7.56E+13 |

Table 10S. Continued

| T | R12 | R13 | R14 | R15 | R16 | R17 |
| --- | --- | --- | --- | --- | --- | --- |
| 298 | 1.02E-40 | 3.23E-50 | 1.50E-59 | 2.47E-51 | 2.26E-50 | 1.35E-50 |
| 300 | 2.69E-40 | 9.69E-50 | 5.06E-59 | 7.41E-51 | 6.72E-50 | 3.98E-50 |
| 400 | 1.57E-24 | 6.82E-32 | 2.61E-39 | 4.50E-33 | 2.97E-32 | 1.39E-32 |
| 500 | 4.75E-15 | 3.83E-21 | 1.95E-27 | 2.41E-22 | 1.31E-21 | 5.22E-22 |
| 600 | 1.00E-08 | 5.88E-14 | 1.71E-19 | 3.69E-15 | 1.76E-14 | 6.26E-15 |
| 700 | 3.29E-04 | 8.10E-09 | 8.36E-14 | 5.25E-10 | 2.26E-09 | 7.44E-10 |
| 800 | 7.90E-01 | 5.81E-05 | 1.59E-09 | 3.96E-06 | 1.58E-05 | 4.88E-06 |
| 900 | 3.33E+02 | 5.81E-02 | 3.43E-06 | 4.20E-03 | 1.58E-02 | 4.64E-03 |
| 1000 | 4.13E+04 | 1.46E+01 | 1.62E-03 | 1.12E+00 | 3.99E+00 | 1.13E+00 |
| 1100 | 2.10E+06 | 1.33E+03 | 2.51E-01 | 1.08E+02 | 3.72E+02 | 1.02E+02 |
| 1200 | 5.49E+07 | 5.69E+04 | 1.69E+01 | 4.95E+03 | 1.64E+04 | 4.38E+03 |
| 1300 | 8.59E+08 | 1.36E+06 | 5.99E+02 | 1.26E+05 | 4.05E+05 | 1.06E+05 |
| 1400 | 8.97E+09 | 2.06E+07 | 1.28E+04 | 2.02E+06 | 6.36E+06 | 1.62E+06 |
| 1500 | 6.80E+10 | 2.16E+08 | 1.82E+05 | 2.24E+07 | 6.90E+07 | 1.74E+07 |
| 1600 | 3.96E+11 | 1.68E+09 | 1.86E+06 | 1.85E+08 | 5.58E+08 | 1.38E+08 |
| 1700 | 1.86E+12 | 1.02E+10 | 1.45E+07 | 1.19E+09 | 3.51E+09 | 8.62E+08 |
| 1800 | 7.33E+12 | 5.09E+10 | 9.02E+07 | 6.21E+09 | 1.82E+10 | 4.40E+09 |
| 1900 | 2.49E+13 | 2.12E+11 | 4.63E+08 | 2.73E+10 | 7.86E+10 | 1.89E+10 |
| 2000 | 7.39E+13 | 7.66E+11 | 2.02E+09 | 7.29E+10 | 2.95E+11 | 7.00E+10 |

Ref. 30

(30) Frisch, M. J.; Trucks, G. W.; Schlegel, H. B.; Scuseria, G. E.; Robb, M. A.; Cheeseman, J. R.; Scalmani, G.; Barone, V.; Mennucci, B.; Petersson, G. A.; Nakatsuji, H.; Caricato, M.; Li, X.; Hratchian, H. P.; Izmaylov, A. F.; Bloino, J.; Zheng, G.; Sonnenberg, J. L.; Hada, M.; Ehara, M.; Toyota, K.; Fukuda, R.; Hasegawa, J.; Ishida, M.; Nakajima, T.; Honda, Y.; Kitao, O.; Nakai, H.; Vreven, T.; Montgomery Jr., J. A.; Peralta, J. E.; Ogliaro, F.; Bearpark, M.; Heyd, J. J.; Brothers, E.; Kudin, K. N.; Staroverov, V. N.; Kobayashi, R.; Normand, J.; Raghavachari, K.; Rendell, A.; Burant, J. C.; Iyengar, S. S.; Tomasi, J.; Cossi, M.; Rega, N.; Millam, J. M.; Klene, M.; Knox, J. E.; Cross, J. B.; Bakken, V.; Adamo, C.; Jaramillo, J.; Gomperts, R.; Stratmann, R. E.; Yazyev, O.; Austin, A. J.; Cammi, R.; Pomelli, C.; Ochterski, J. W.; Martin, R. L.; Morokuma, K.; Zakrzewski, V. G.; Voth, G. A.; Salvador, P.; Dannenberg, J. J.; Dapprich, S.; Daniels, A. D.; Farkas, O.; Foresman, J. B.; Ortiz, J. V.; Cioslowski, J.; Fox, D. J. *Gaussian 09*; Gaussian, Inc.: Wallingford, CT, 2009.
